# Low Diversity *Cryptococcus neoformans* Variety *grubii* Multilocus Sequence Types from Thailand Are Consistent with an Ancestral African Origin

**DOI:** 10.1371/journal.ppat.1001343

**Published:** 2011-04-28

**Authors:** Sitali P. Simwami, Kantarawee Khayhan, Daniel A. Henk, David M. Aanensen, Teun Boekhout, Ferry Hagen, Annemarie E. Brouwer, Thomas S. Harrison, Christl A. Donnelly, Matthew C. Fisher

**Affiliations:** 1 Department of Infectious Disease Epidemiology, Faculty of Medicine, Imperial College London, London, United Kingdom; 2 CBS Fungal Biodiversity Centre, Utrecht, The Netherlands; 3 Department of Microbiology and Parasitology, School of Medical Science, Naresuan University Phayao, Phayao, Thailand; 4 Department of General Internal Medicine and Nijmegen University Center for Infectious Diseases, Radboud University Medical Centre, Nijmegen, The Netherlands; 5 St. Elisabeth Hospital, Tilburg, The Netherlands; 6 Department of Infectious Diseases, St George's Hospital Medical School, London, United Kingdom; Duke University Medical Center, United States of America

## Abstract

The global burden of HIV-associated cryptococcal meningitis is estimated at nearly one million cases per year, causing up to a third of all AIDS-related deaths. Molecular epidemiology constitutes the main methodology for understanding the factors underpinning the emergence of this understudied, yet increasingly important, group of pathogenic fungi. *Cryptococcus* species are notable in the degree that virulence differs amongst lineages, and highly-virulent emerging lineages are changing patterns of human disease both temporally and spatially. *Cryptococcus neoformans* variety *grubii* (*Cng*, serotype A) constitutes the most ubiquitous cause of cryptococcal meningitis worldwide, however patterns of molecular diversity are understudied across some regions experiencing significant burdens of disease. We compared 183 clinical and environmental isolates of *Cng* from one such region, Thailand, Southeast Asia, against a global MLST database of 77 *Cng* isolates. Population genetic analyses showed that Thailand isolates from 11 provinces were highly homogenous, consisting of the same genetic background (globally known as VNI) and exhibiting only ten nearly identical sequence types (STs), with three (STs 44, 45 and 46) dominating our sample. This population contains significantly less diversity when compared against the global population of *Cng*, specifically Africa. Genetic diversity in *Cng* was significantly subdivided at the continental level with nearly half (47%) of the global STs unique to a genetically diverse and recombining population in Botswana. These patterns of diversity, when combined with evidence from haplotypic networks and coalescent analyses of global populations, are highly suggestive of an expansion of the *Cng* VNI clade out of Africa, leading to a limited number of genotypes founding the Asian populations. Divergence time testing estimates the time to the most common ancestor between the African and Asian populations to be 6,920 years ago (95% HPD 122.96 - 27,177.76). Further high-density sampling of global *Cng* STs is now necessary to resolve the temporal sequence underlying the global emergence of this human pathogen.

## Author Summary

*Cryptococcus neoformans* is a species complex of often highly pathogenic fungi that cause significant disease in humans. *Cryptococcus* is notable in the degree that virulence differs amongst genotypes, and highly-virulent emerging lineages are changing patterns of disease in time and space. *Cryptococcus neoformans* variety *grubii* (*Cng*) causes meningitis among HIV/AIDS patients, up to 1 million cases/year resulting in over 600,000 mortalities. Despite these rates of mortality being comparable to those caused by malaria (one million mortalities per annum), cryptococcal meningitis receives only a fraction of the attention, funding and control granted to more widely recognised diseases. This study uses multilocus sequence typing to compare the genetic diversity of *Cng* in a largely unstudied country with an emerging HIV epidemic, Thailand, against the diversity seen elsewhere. We found that *Cng* in Thailand exhibits significantly less genetic diversity in comparison to other areas of the world, especially Africa. Analyses dating the pathogen's origin in Thailand support the introduction of a limited number of genotypes into Southeast Asia from an ancestral African population within the last 7,000 years. These findings show the power associated with the collection of global sequence databases in order to better understand the evolution of major fungal pathogens.

## Introduction

*Cryptococcus neoformans* (*Cn*) is an encapsulated basidiomycetous yeast, and the etiological agent of the invasive fungal infection cryptococcosis. The first clinical discovery of *Cn* was in 1894, and this pathogen has since become one of the leading causes of mycotic morbidity and mortality worldwide [1], [2], [3]. Capable of causing disease among both immunocompetent and immunocompromised individuals, the most common manifestation of cryptococcosis is cryptococcal meningitis (CM) [4], [5]. The HIV/AIDS epidemic has driven increased *Cryptococcus* infection rates via the rapid increase of immunosuppressed populations [1], [6], [7]. Patients with HIV-related CM must undergo maintenance anti-fungal therapy life-long or until immunoreconstitution is reached by antiretroviral therapy [8], and mortality rates remain unacceptably high [3].

Originally believed to be a single species, two distinct varieties of *Cn* have been described, corresponding to three serotypes: *Cn* var *grubii* (serotype A; henceforth *Cng*), *Cn* var *neoformans* (serotype D) and AD hybrids [9]. *C. gattii*, a second species of the genus *Cryptococcus*, consists of serotypes B and C [10], and is also capable of forming hybrids with *Cn* [11], [12], [13]. Molecular typing has resulted in these two species being further subdivided into eight major molecular types: VNI and VNII (serotype A; var *grubii*), VNIII (hybrid serotype AD; var *neoformans*), VNIV (serotype D; var *neoformans*), VGI, VGII, VGIII and VGIV (serotypes B and C; var *gattii*) [12], [13], [14], [15]. Within *Cng*, VNI predominates worldwide, including in Southeast Asian countries such as Thailand [16] and Malaysia [17]. *Cn* has two mating types, *MAT*α and *MAT* **a**, controlled by a single locus, two allele mating system [18]. Globally, there is a predominance of mating-type *MAT*α among both environmental and clinical samples across serotypes [19], [20], [21], [22], [23]. An exception is the less common AD hybrid, 68% of which possess the *MAT*a allele from serotype A as well as the *MAT*α allele from serotype D [19]. This discrepancy in mating type prevalence is also observed in other pathogenic fungi including *Histoplasma capsulatum* and several species of dermatophyte fungi [24], [25], [26], [27], [28].

*Cng* (serotype A) is widely associated with avian excreta and other organic substrates [29], [30], [31], [32], and is known to infect mainly immunocompromised hosts [1], [33], although there has been evidence of cryptococcosis due to *Cng* among patients with no underlying disease [34], [35], [36]. Distributed nearly worldwide and commonly isolated from the environment, this variety is responsible for about 95% of cryptococcal infections worldwide [31] and 98% of infections among AIDS patients [6]. However, despite the emerging importance of this pathogen and increased research effort [13], [37], aspects of the pathogen's global population genetic structure remain undetermined. This is especially true for Southeast Asia where cryptococcosis affects nearly 20% of HIV infected patients [38] in this highly populous region.

An accurate description of the genetic composition of fungal pathogen populations is important from several standpoints: quantifying the amount and distribution of polymorphisms across space and time enables the identification of population-level processes that ultimately lead to an understanding of the process of infection, such as the reservoirs, transmissibility and longevity of populations and their component genotypes. Increasingly, it is being recognised that specific genotypes act as markers of lineages that exhibit enhanced or reduced virulence [39], [40], [41], [42]. Therefore, an accurate understanding of the genetics of these pathogens clarifies their current and future evolutionary trajectories, and their potential to alter the burden of human disease.

To accurately discriminate between isolates of *Cng* and to enable the rapid acquisition of global genotypic data, the International Society of Human and Animal Mycoses (ISHAM) special working group on *Cryptococcus* and cryptococcosis recognized the need for a cross-platform consensus-typing scheme for *Cn*. This typing scheme needed to be able to incorporate the findings from previous global-typing projects, while being universally applicable, publicly available and able to integrate new data as they emerged. Previously, PCR fingerprinting with the minisatellite-specific core sequence of the wild-type phage M13 or microsatellites was utilized in local-scale studies on patterns of genetic diversity, identifying three major molecular types of *Cng*, VNI, VNII and VNB [37], [43]. The ISHAM group has selected multi-locus sequence typing (MLST) using seven loci as the method of choice for global molecular epidemiological typing of *Cryptococcus* species *Cng* [44]. The molecular type (VN system) [15] has been maintained as the standardized naming system for specific related clades of sequence types (STs). Using MLST-approaches, Litvintseva *et al*. (2006) have demonstrated marked heterogeneity in the global distribution of VN-types with a highly genetically diverse, area-specific and recombining population of VNB genotypes in Africa (Botswana) [37].

Increasingly, it is recognised that many human infectious diseases have emerged within the last 11,000 years, following the rise of agriculture and domestication of animals [45]. The consequential globalisation of microbes that have been carried along with this human expansion has left its mark in the population genetic structure of both transmissible [46] and non-transmissible environmental pathogens [47]. One such pathogen is the sister species of *Cng*, *C. gattii*, which has seen a rapid rise in human infections in the non-tropical Pacific Northwest areas of Canada and the United States. Here the introduction of *C. gattii* is believed to have occurred more recently, perhaps vectored by the international trade in Eucalyptus trees from Australia where the species is most commonly found [40], [42], [48]. The discovery of a population displaying ancestral characteristics in southern Africa, and a global distribution of clonally-derived and genetically homogenous VNI genotypes [37], has led Litvintseva *et al*, 2006 to hypothesise that *Cng* has an evolutionary origin in Africa followed by a global expansion, possibly vectored by the migration of avian species (conference abstract, Fungal Genetics Reports: 56S). The common pigeon (*Columba livia*), originating in Africa, is considered a mechanistic carrier and potential spreader of the fungus, its faeces being a common environmental source of *Cng* [49], [50], [51]. Although unable to systemically colonize these birds, *Cng* can survive the elevated temperatures within their gastrointestinal tract (41 - 42°C), as well as remain alive for up to two years in the birds' excreta [50]. These birds were domesticated in Africa approximately 5,000 years ago and introduced to Europe, then subsequently distributed to many parts of the world during the European expansion in the last 500 years [52], [53]; a range expansion that may have led to pigeon vectors allowing *Cng* to broaden its global ecological range. While wind transport has also been hypothesized as a potential method of the global dispersal of *Cng*, as demonstrated by the potential for dispersal of *Coccidioides immitis* by wind-blown arthroconidia [54], Casadeval and Perfect state that this is unlikely, due to the *Cng* basidiospores being unsuitable for long-distance wind dispersal [31].

The aim of this study was to describe the population genetic structure of the previously untyped, but clinically important, population of *Cng* that infects HIV/AIDS patients in Thailand, Southeast Asia, with the intention of integrating these data into broader global patterns. Our specific goals were (i) to describe the genetic structure of this population of *Cng* using MLST, (ii) to compare the population genetic structure of these isolates against the global collection of *Cng* STs and (iii) to investigate potential associations between infecting genotypes of *Cng* and disease progression among HIV-AIDS patients.

## Results

### Mating-type and serotypes of *Cng* isolates

All 183 Thai isolates typed in this study were *Cng (*serotype A) and of mating type MATα. Ten were from environmental sources in Chiang Mai, Northern Thailand, while 83 of the 173 clinical isolates (48%) originated from the North, 78 from the Northeast (45%) and 9 (5%) from the South of Thailand (three were of unknown origin; table 1). All 77 of the global isolates were also *Cng*. Thirteen percent of these (*n* = 10) were of mating type MAT**a**, nine originating from Botswana, and one from Tanzania (table S1) [41]. Previously typed by both Amplified Fragment Length Polymorphism (AFLP) and MLST, three molecular groups within serotype A were present in the global isolates: VNI  = 48 (62%), VNII  = 9 (12%) and VNB  = 20 (26%) [37].

**Table 1:** The allelic profiles of the 183 *Cn*g isolates from Thailand typed by MLST in this study.

Name	CAP59 allele(501 bp)	GPD1allele(489 bp)	IGS1 allele(709 bp)	LAC1 allele(471 bp)	PLB1 allele(533 bp)	SOD1 allele(527 bp)	URA5 allele(637 bp)	ST	Strain origin (if known)
**CN5010**	1	1	**19**	3	2	**13**	5	44	Chiang Rai, Thailand, blood
**CN4998**	1	1	**19**	3	2	**13**	5	44	Chiang Mai, Thailand, CSF
**CN4995**	1	1	**19**	3	2	**13**	5	44	Chiang Mai, Thailand, CSF
**CN4989**	1	1	**19**	3	2	**13**	5	44	Chiang Mai, Thailand, CSF
**CN4988**	1	1	**19**	3	2	**13**	5	44	Chiang Mai, Thailand, CSF
**CN4987**	1	1	**19**	3	2	**13**	5	44	Chiang Mai, Thailand, CSF
**CN4964**	1	1	**19**	3	2	**13**	5	44	Chiang Mai, Thailand, CSF
**CN4947**	1	1	**19**	3	2	**13**	5	44	Chiang Rai, Thailand, CSF
**CN4945**	1	1	**19**	3	2	**13**	5	44	Chiang Rai, Thailand, CSF
**CN4944**	1	1	**19**	3	2	**13**	5	44	Chiang Mai, Thailand, CSF
**CN4943**	1	1	**19**	3	2	**13**	5	44	Chiang Rai, Thailand
**CN4942**	1	1	**19**	3	2	**13**	5	44	Lampang, Thailand, CSF
**CN4941**	1	1	**19**	3	2	**13**	5	44	Thailand, CSF
**CN4940**	1	1	**19**	3	2	**13**	5	44	Thailand, CSF
**CN4926**	1	1	**19**	3	2	**13**	5	44	Chiang Rai, Thailand, CSF
**CN4919**	1	1	**19**	3	2	**13**	5	44	Chiang Rai, Thailand, CSF
**CN4918**	1	1	**19**	3	2	**13**	5	44	Chiang Rai, Thailand, CSF
**CN4917**	1	1	**19**	3	2	**13**	5	44	Chiang Rai, Thailand, CSF
**CN4903**	1	1	**19**	3	2	**13**	5	44	Chiang Rai, Thailand, CSF
**CN4901**	1	1	**19**	3	2	**13**	5	44	Chiang Mai, Thailand, CSF
**CN49005**	1	1	**19**	3	2	**13**	5	44	Chiang Mai, Thailand
**4-187**	1	1	**19**	3	2	**13**	5	44	Khon Kaen, Thailand, clinical
**269**	1	1	**19**	3	2	**13**	5	44	Khon Kaen, Thailand, clinical
**4-315**	1	1	**19**	3	2	**13**	5	44	Khon Kaen, Thailand, clinical
**1-587**	1	1	**19**	3	2	**13**	5	44	Khon Kaen, Thailand, clinical
**1219**	1	1	**19**	3	2	**13**	5	44	Khon Kaen, Thailand, clinical
**4_83**	1	1	**19**	3	2	**13**	5	44	Khon Kaen, Thailand, clinical
**1-588**	1	1	**19**	3	2	**13**	5	44	Khon Kaen, Thailand, clinical
**4-202**	1	1	**19**	3	2	**13**	5	44	Khon Kaen, Thailand, clinical
**1-846**	1	1	**19**	3	2	**13**	5	44	Khon Kaen, Thailand, clinical
**2551-07**	1	1	**19**	3	2	**13**	5	44	Songkhla, Thailand, CSF
**2550 II-07**	1	1	**19**	3	2	**13**	5	44	Songkhla, Thailand, blood
**2461-07**	1	1	**19**	3	2	**13**	5	44	Songkhla, Thailand, CSF
**CM 1**	1	1	**19**	3	2	**13**	5	44	Ubon Ratchathani, Thailand, CSF
**CM 6**	1	1	**19**	3	2	**13**	5	44	Ubon Ratchathani, Thailand, CSF
**CM 7**	1	1	**19**	3	2	**13**	5	44	Ubon Ratchathani, Thailand, CSF
**CM 8**	1	1	**19**	3	2	**13**	5	44	Ubon Ratchathani, Thailand, CSF
**CM 12**	1	1	**19**	3	2	**13**	5	44	Ubon Ratchathani, Thailand, CSF
**CM 13**	1	1	**19**	3	2	**13**	5	44	Ubon Ratchathani, Thailand, CSF
**CM 17**	1	1	**19**	3	2	**13**	5	44	Ubon Ratchathani, Thailand, CSF
**CM 18**	1	1	**19**	3	2	**13**	5	44	Ubon Ratchathani, Thailand, CSF
**CM 22**	1	1	**19**	3	2	**13**	5	44	Ubon Ratchathani, Thailand, CSF
**CM 23**	1	1	**19**	3	2	**13**	5	44	Ubon Ratchathani, Thailand, CSF
**CM 25**	1	1	**19**	3	2	**13**	5	44	Ubon Ratchathani, Thailand, CSF
**CM 26**	1	1	**19**	3	2	**13**	5	44	Ubon Ratchathani, Thailand, CSF
**CM 33**	1	1	**19**	3	2	**13**	5	44	Ubon Ratchathani, Thailand, CSF
**CM 37**	1	1	**19**	3	2	**13**	5	44	Ubon Ratchathani, Thailand, CSF
**CM 38**	1	1	**19**	3	2	**13**	5	44	Ubon Ratchathani, Thailand, CSF
**CM 39**	1	1	**19**	3	2	**13**	5	44	Ubon Ratchathani, Thailand, CSF
**CM 40**	1	1	**19**	3	2	**13**	5	44	Ubon Ratchathani, Thailand, CSF
**CM 41**	1	1	**19**	3	2	**13**	5	44	Ubon Ratchathani, Thailand, CSF
**CM42**	1	1	**19**	3	2	**13**	5	44	Ubon Ratchathani, Thailand, CSF
**CM 43**	1	1	**19**	3	2	**13**	5	44	Ubon Ratchathani, Thailand, CSF
**CM 44**	1	1	**19**	3	2	**13**	5	44	Ubon Ratchathani, Thailand, CSF
**CM 46**	1	1	**19**	3	2	**13**	5	44	Ubon Ratchathani, Thailand, CSF
**CM 47**	1	1	**19**	3	2	**13**	5	44	Ubon Ratchathani, Thailand, CSF
**CM 48**	1	1	**19**	3	2	**13**	5	44	Ubon Ratchathani, Thailand, CSF
**CM 49**	1	1	**19**	3	2	**13**	5	44	Ubon Ratchathani, Thailand, CSF
**CM 51**	1	1	**19**	3	2	**13**	5	44	Ubon Ratchathani, Thailand, CSF
**CM 55**	1	1	**19**	3	2	**13**	5	44	Ubon Ratchathani, Thailand, CSF
**CM 56**	1	1	**19**	3	2	**13**	5	44	Ubon Ratchathani, Thailand, CSF
**CM 57**	1	1	**19**	3	2	**13**	5	44	Ubon Ratchathani, Thailand, CSF
**CM 58**	1	1	**19**	3	2	**13**	5	44	Ubon Ratchathani, Thailand, CSF
**CM 59**	1	1	**19**	3	2	**13**	5	44	Ubon Ratchathani, Thailand, CSF
**CM 61**	1	1	**19**	3	2	**13**	5	44	Ubon Ratchathani, Thailand, CSF
**CM 63**	1	1	**19**	3	2	**13**	5	44	Ubon Ratchathani, Thailand, CSF
**K 2**	1	1	**19**	3	2	**13**	5	44	Khon Kaen, Thailand, crypto patient
**Pg 1**	1	1	**19**	3	2	**13**	5	44	Chiang Mai, Thailand, pigeon dropping
**D 6**	1	1	**19**	3	2	**13**	5	44	Chiang Mai, Thailand, dove dropping
**D 1**	1	1	**19**	3	2	**13**	5	44	Chiang Mai, Thailand, dove dropping
**CN5019**	1	1	**19**	4	2	**13**	5	45	Chiang Rai, Thailand, blood
**CN5017**	1	1	**19**	4	2	**13**	5	45	Chiang Rai, Thailand, CSF
**CN5014**	1	1	**19**	4	2	**13**	5	45	Chiang Rai, Thailand, blood
**CN5013**	1	1	**19**	4	2	**13**	5	45	Chiang Rai, Thailand, CSF
**CN5011**	1	1	**19**	4	2	**13**	5	45	Thailand, clinical
**CN5009**	1	1	**19**	4	2	**13**	5	45	Chiang Rai, Thailand, blood
**CN5005**	1	1	**19**	4	2	**13**	5	45	Chiang Rai, Thailand, blood
**CN5003**	1	1	**19**	4	2	**13**	5	45	Chiang Rai, Thailand, blood
**CN5002**	1	1	**19**	4	2	**13**	5	45	Chiang Rai, Thailand, blood
**CN5001**	1	1	**19**	4	2	**13**	5	45	Chiang Rai, Thailand, CSF
**CN4970**	1	1	**19**	4	2	**13**	5	45	Chiang Mai, Thailand, CSF
**CN4968**	1	1	**19**	4	2	**13**	5	45	Chiang Mai, Thailand, CSF
**CN4957**	1	1	**19**	4	2	**13**	5	45	Chiang Rai, Thailand, CSF
**CN4956**	1	1	**19**	4	2	**13**	5	45	Chiang Rai, Thailand, CSF
**CN4955**	1	1	**19**	4	2	**13**	5	45	Thailand, BAL
**CN4954**	1	1	**19**	4	2	**13**	5	45	Lampang, Thailand, CSF
**CN4952**	1	1	**19**	4	2	**13**	5	45	Tak, Thailand, CSF
**CN4950**	1	1	**19**	4	2	**13**	5	45	Lampoon, Thailand, CSF
**CN4949**	1	1	**19**	4	2	**13**	5	45	Lampoon, Thailand, CSF
**CN4938**	1	1	**19**	4	2	**13**	5	45	Chiang Mai, Thailand, CSF
**CN4937**	1	1	**19**	4	2	**13**	5	45	Chiang Mai, Thailand, CSF
**CN4936**	1	1	**19**	4	2	**13**	5	45	Chiang Mai, Thailand, CSF
**CN4934**	1	1	**19**	4	2	**13**	5	45	Chiang Mai, Thailand, CSF
**CN4933**	1	1	**19**	4	2	**13**	5	45	Chiang Mai, Thailand, CSF
**CN4932**	1	1	**19**	4	2	**13**	5	45	Chiang Mai, Thailand, CSF
**CN4931**	1	1	**19**	4	2	**13**	5	45	Chiang Mai, Thailand, CSF
**CN4927**	1	1	**19**	4	2	**13**	5	45	Chiang Mai, Thailand, CSF
**CN4915**	1	1	**19**	4	2	**13**	5	45	Chiang Mai, Thailand, CSF
**CN4914**	1	1	**19**	4	2	**13**	5	45	Chiang Mai, Thailand, CSF
**CN4909**	1	1	**19**	4	2	**13**	5	45	Chiang Mai, Thailand, CSF
**CN4907**	1	1	**19**	4	2	**13**	5	45	Chiang Mai, Thailand, CSF
**CN4905**	1	1	**19**	4	2	**13**	5	45	Chiang Mai, Thailand, CSF
**CN4904**	1	1	**19**	4	2	**13**	5	45	Chiang Mai, Thailand, CSF
**CN4902**	1	1	**19**	4	2	**13**	5	45	Chiang Mai, Thailand, CSF
**CN49008**	1	1	**19**	4	2	**13**	5	45	Chiang Mai, Thailand, CSF
**4-319**	1	1	**19**	4	2	**13**	5	45	Khon Kaen, Thailand, clinical
**50NC2**	1	1	**19**	4	2	**13**	5	45	Nan, Thailand, clinical
**50NC5**	1	1	**19**	4	2	**13**	5	45	Nan, Thailand, clinical
**11112**	1	1	**19**	4	2	**13**	5	45	Khon Kaen, Thailand, clinical
**11109**	1	1	**19**	4	2	**13**	5	45	Khon Kaen, Thailand, clinical
**4-231**	1	1	**19**	4	2	**13**	5	45	Khon Kaen, Thailand, clinical
**P6**	1	1	**19**	4	2	**13**	5	45	Chiang Mai, Thailand, clinical
**4-253**	1	1	**19**	4	2	**13**	5	45	Khon Kaen, Thailand, clinical
**4-381**	1	1	**19**	4	2	**13**	5	45	Khon Kaen, Thailand, clinical
**20662-07**	1	1	**19**	4	2	**13**	5	45	Songkhla, Thailand, blood
**28170-07**	1	1	**19**	4	2	**13**	5	45	Songkhla, Thailand, CSF
**1111I-08**	1	1	**19**	4	2	**13**	5	45	Pattani, Thailand, blood/HIV-
**2895I-08**	1	1	**19**	4	2	**13**	5	45	Pattani, Thailand, blood/HIV-
**4500-07**	1	1	**19**	4	2	**13**	5	45	Pattani, Thailand, blood
**CM 2**	1	1	**19**	4	2	**13**	5	45	Ubon Ratchathani, Thailand, CSF
**CM 3**	1	1	**19**	4	2	**13**	5	45	Ubon Ratchathani, Thailand, CSF
**CM 4**	1	1	**19**	4	2	**13**	5	45	Ubon Ratchathani, Thailand, CSF
**CM 5**	1	1	**19**	4	2	**13**	5	45	Ubon Ratchathani, Thailand, CSF
**CM 10**	1	1	**19**	4	2	**13**	5	45	Ubon Ratchathani, Thailand, CSF
**CM 14**	1	1	**19**	4	2	**13**	5	45	Ubon Ratchathani, Thailand, CSF
**CM 11**	1	1	**19**	4	2	**13**	5	45	Ubon Ratchathani, Thailand, CSF
**CM 15**	1	1	**19**	4	2	**13**	5	45	Ubon Ratchathani, Thailand, CSF
**CM16**	1	1	**19**	4	2	**13**	5	45	Ubon Ratchathani, Thailand, CSF
**CM 20**	1	1	**19**	4	2	**13**	5	45	Ubon Ratchathani, Thailand, CSF
**CM 24**	1	1	**19**	4	2	**13**	5	45	Ubon Ratchathani, Thailand, CSF
**CM 27**	1	1	**19**	4	2	**13**	5	45	Ubon Ratchathani, Thailand, CSF
**CM 28**	1	1	**19**	4	2	**13**	5	45	Ubon Ratchathani, Thailand, CSF
**CM 29**	1	1	**19**	4	2	**13**	5	45	Ubon Ratchathani, Thailand, CSF
**CM 32**	1	1	**19**	4	2	**13**	5	45	Ubon Ratchathani, Thailand, CSF
**CM 34**	1	1	**19**	4	2	**13**	5	45	Ubon Ratchathani, Thailand, CSF
**CM 36**	1	1	**19**	4	2	**13**	5	45	Ubon Ratchathani, Thailand, CSF
**CM 45**	1	1	**19**	4	2	**13**	5	45	Ubon Ratchathani, Thailand, CSF
**CM 50**	1	1	**19**	4	2	**13**	5	45	Ubon Ratchathani, Thailand, CSF
**CM 52**	1	1	**19**	4	2	**13**	5	45	Ubon Ratchathani, Thailand, CSF
**CM 60**	1	1	**19**	4	2	**13**	5	45	Ubon Ratchathani, Thailand, CSF
**CM 64**	1	1	**19**	4	2	**13**	5	45	Ubon Ratchathani, Thailand, CSF
**Pt 9**	1	1	**19**	4	2	**13**	5	45	Chiang Mai, Thailand, crypto patient
**Pt 3**	1	1	**19**	4	2	**13**	5	45	Chiang Mai, Thailand, crypto patient
**Pt 1**	1	1	**19**	4	2	**13**	5	45	Chiang Mai, Thailand, crypto patient
**D 2**	1	1	**19**	4	2	**13**	5	45	Chiang Mai, Thailand, dove dropping
**D 3**	1	1	**19**	4	2	**13**	5	45	Chiang Mai, Thailand, dove dropping
**Pg 2**	1	1	**19**	4	2	**13**	5	45	Chiang Mai, Thailand, pigeon dropping
**Pg 26**	1	1	**19**	4	2	**13**	5	45	Chiang Mai, Thailand, pigeon dropping
**CN49004**	1	3	**19**	5	2	**13**	1	46	Chiang Mai, Thailand, CSF
**CN48**	1	3	**19**	5	2	**13**	1	46	Khon Kaen, Thailand, clinical
**1-488**	1	3	**19**	5	2	**13**	1	46	Khon Kaen, Thailand, clinical
**1-489**	1	3	**19**	5	2	**13**	1	46	Khon Kaen, Thailand, clinical
**CM 30**	1	3	**19**	5	2	**13**	1	46	Ubon Ratchathani, Thailand, CSF
**Pt 12**	1	3	**19**	5	2	**13**	1	46	Chiang Mai, Thailand, crypto patient
**D 5**	1	3	**19**	5	2	**13**	1	46	Chiang Mai, Thailand, dove dropping
**Pg 37**	1	3	**19**	5	2	**13**	1	46	Chiang Mai, Thailand, pigeon dropping
**CN5015**	1	3	**19**	5	2	**13**	1	46	Chiang Rai, Thailand, CSF
**CN5018**	1	3	**19**	5	2	**13**	1	46	Chiang Rai, Thailand, blood
**CN5012**	1	3	**19**	5	2	**13**	1	46	Chiang Rai, Thailand, CSF
**CN5008**	1	3	**19**	5	2	**13**	1	46	Chiang Rai, Thailand, CSF
**CN4993**	1	3	**19**	5	2	**13**	1	46	Chiang Mai, Thailand, CSF
**CN4983**	1	3	**19**	5	2	**13**	1	46	Chiang Mai, Thailand, CSF
**CN4980**	1	3	**19**	5	2	**13**	1	46	Chiang Mai, Thailand, CSF
**CN4977**	1	3	**19**	5	2	**13**	1	46	Chiang Mai, Thailand, CSF
**CN4967**	1	3	**19**	5	2	**13**	1	46	Chiang Mai, Thailand, CSF
**CN4960**	1	3	**19**	5	2	**13**	1	46	Chiang Rai, Thailand, CSF
**CN4948**	1	3	**19**	5	2	**13**	1	46	Chiang Mai, Thailand, CSF
**CN4946**	1	3	**19**	5	2	**13**	1	46	Chiang Mai, Thailand, CSF
**CN4924**	1	3	**19**	5	2	**13**	1	46	Chiang Mai, Thailand, CSF
**CN4921**	1	3	**19**	5	2	**13**	1	46	Mae Hong Son, Thailand, CSF
**CN4920**	1	3	**19**	5	2	**13**	1	46	Chiang Mai, Thailand, CSF
**CN4916**	1	3	**19**	5	2	**13**	1	46	Chiang Mai, Thailand, CSF
**CN4906**	1	3	**19**	5	2	**13**	1	46	Chiang Mai, Thailand, CSF
**CN49006**	1	3	**19**	5	2	**13**	1	46	Chiang Mai, Thailand, CSF
**50NC1**	1	3	**19**	**10**	2	**13**	1	53	Nan, Thailand, clinical
**Pt 5**	1	1	**19**	5	2	**13**	1	51	Chiang Mai, Thailand, crypto patient
**CN5007**	1	1	**20**	3	4	**13**	1	47	Chiang Rai, Thailand, CSF
**1291-09**	1	1	**20**	3	4	**13**	1	47	Pattani, Thailand, blood/HIV-
**CM 35**	1	1	**20**	3	4	**13**	1	47	Ubon Ratchathani, Thailand, CSF
**K 45**	1	1	**19**	3	4	**13**	5	50	Khon Kaen, Thailand, crypto patient
**4_9**	1	1	**19**	**9**	2	**13**	5	52	Khon Kaen, Thailand, clinical
**D 9**	1	1	**19**	4	2	**13**	**14**	49	Chiang Mai, Thailand, dove dropping
**CM 21**	2	10	**21**	6	11	**14**	4	48	Ubon Ratchathani, Thailand, CSF

bp  =  base pairs; crypto patient  =  cryptococcosis patient; novel ATs are in bold.

### MLST determination

Sequence data were obtained for all 183 Thai isolates typed at the seven loci (table 1). The aligned sequences of the concatenated loci were 3,959 base pairs in total, with 112 polymorphic sites (20 parsimony informative and 92 singleton sites). The seven loci yielded 23 allele types (ATs), eight of which were novel to the Thai population of *Cng* (table 1). Loci *IGS1* and *SOD1* consisted entirely of novel ATs, while *CAP59*, *GPD1* and *PLB1* were made up of previously described ATs [37]. We identified 10 multilocus sequence types (STs) within the Thai isolates.

The collection of 77 global isolates of *Cng* yielded 86 ATs and 43 STs. The concatenated sequences were 3,970 base pairs in length, with 190 variable sites. The ten new STs described in Thailand were allocated consecutive numbers ST 44-53 (table 1), resulting in a complete dataset of 53 global STs for *Cng* (table S1). ST44 accounted for 38% of the Thai isolates (*n* = 70), ST45 for 43% (*n* = 78) and ST46 for 14% (*n* = 26) (table 1). STs 44 and 45 collectively contained 81% of all the isolates and differed only at the *LAC1* gene (nucleotide positions 36, 190, 232 and 338). STs 48 to 53 consisted of single isolates, all of which differed from at least one other ST at a single locus. Nine of the ten environmental isolates shared identical genotypes with clinical isolates.

### Analyses of genetic variation and phylogeny reveal a genetically depauperate Thai *Cng* population

**Figure 1:**
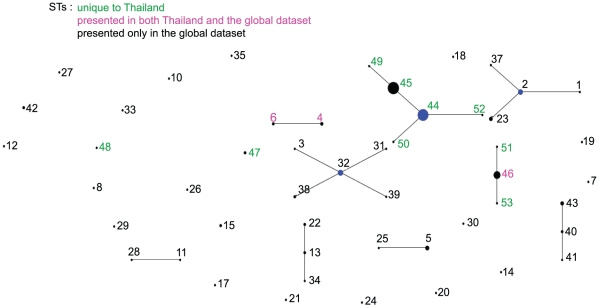
eBURST illustration comparing the isolates from Thailand with the global population of *Cng* used in this study. No. isolates = 176, no. STs = 53, no. re-samplings for bootstrapping = 1000, no. loci per isolate = 7, no. identical loci for group def = 1, no. groups = 1. STs identified by eBURST as present in Thailand and elsewhere in the global dataset are highlighted pink text, those only found in Thailand highlighted green and those only in the global population and not in Thailand are black. Founding genotypes are in blue, and the size of the dots are representative of the number of isolates of that ST.

Initial analyses using eBURST, a web-enabled clustering tool at http://cneoformans.mlst.net/, revealed spatial differentiation between the Thai *Cng* population when compared to the current global population (figure 1). This tool infers patterns of evolutionary descent among clusters of related genotypes from MLST data and identifies mutually exclusive groups of related genotypes within populations. Widespread relatedness was demonstrated within Thailand, shown by the grouping of the majority of Thai STs into a single eBURST group linked by single-locus variants (SLVs; ST44, 45, 49, 50 and 52). STs identified by eBURST as present both in Thailand and elsewhere in the global dataset were highlighted (pink text; ST4, 6, 46; figure 1) and those only found in Thailand shown in green (ST44, 45, 47, 48, 49, 50, 51, 52, 53).

The average nucleotide diversity within the Thai population was explored at all seven loci using haplotypic diversity (*H_d_*), the number of nucleotide differences per site (*π*) and Watterson's estimate of the population scaled mutation rate (*θ*). The average estimates of these statistics for the concatenated sequences were low (*Hd* = 0.19, *π* = 0.001 and *θ* = 0.005 respectively; table S2), reflecting the low number of haplotypes which ranged from two to six at the seven loci. Locus *LAC1,* 467 base pairs long, had the greatest number of segregating sites (*n* = 61), while *CAP59* had the lowest haplotypic diversity and population scaled mutation rate (0.01 and 0.002, respectively).

The spatial partitioning of genetic variability in the Thai *Cng* population typed in this study (*n* = 183) was examined using Analysis of Molecular Variance (AMOVA). This analysis demonstrated that only a small proportion, 5% (*p*<0.013), of the total estimated variance was attributable to the among-population variance component between the three Thai regions (table 2).

**Table 2:** Summary of AMOVA of *Cng* isolates, based on the seven polymorphic loci and according to geographical origin.

	d.f.	Sum of squares	Variance components (%)	ΦPT	*P* - value^a^
(i) Thai population: North (*n* = 92), Northeast (*n* = 78), South (*n* = 9)					
Among populations	2	4	0.03 (5)	0.05	0.013
Within populations	176	114	0.65 (95)		
Total	178	118	0.68 (100)		
(ii) Asian and Global populations: Asia (*n* = 191), Global (*n* = 70)					
Among populations	1	12	1.22 (49)	0.49	0.010
Within populations	259	333	1.28 (51)		
Total	260	459	2.51 (100)		
(iii) Global population^b^: Africa (*n* = 44), Asia (*n* = 191), North America (*n* = 19), South America (*n* = 5)					
Among populations	3	145	1.29 (52)	0.52	0.001
Within populations	255	308	1.21 (48)		
Total	258	452	2.5 (100)		

a *P* - value estimates are based on 999 permutations.

b Europe was excluded due to small sample size (*n* = 2).

**Figure 2:**
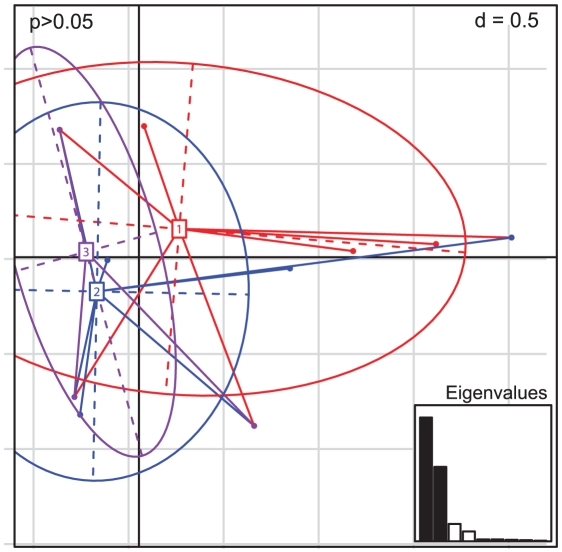
Principle Components Analysis of the allelic profiles of the Thai *Cng* genotypes typed in this study. Individual genotypes (dots) are linked by coloured lines to form clusters which are summarised by coloured ellipses proportional in size to the number of isolates represented. The three groups depicted are numbered and defined according to Thai region: 1 = North (red; *n* = 91), 2 = Northeast (blue; *n* = 79) and 3 = South (purple; *n* = 9). *P* - value is shown and eigenvalues represented in the bar plot.

**Figure 3:**
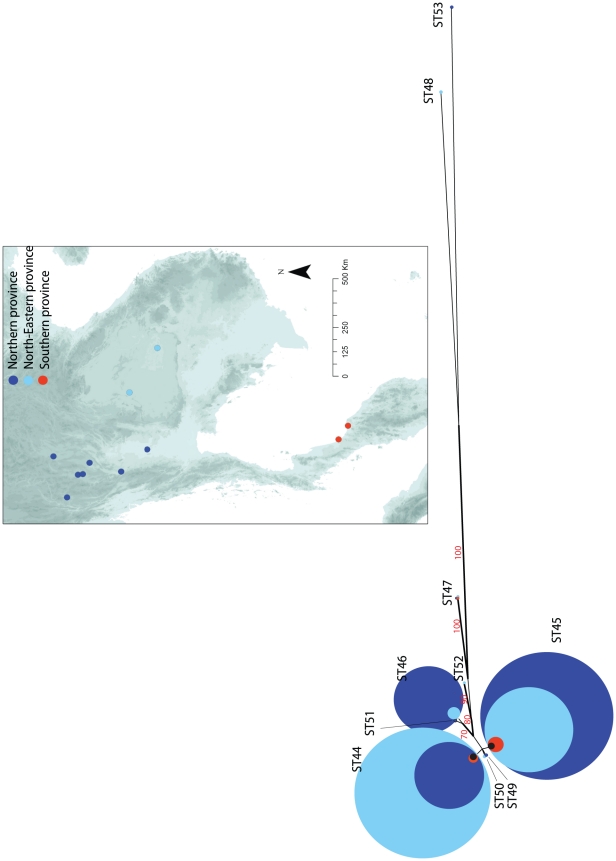
Neighbour-joining tree inferring the evolutionary relationships of the Thai isolates typed in this study (*n* = 183). Each circle represents a Sequence Type (ST) of the Thai isolates and is proportional in size to the number of isolates of this ST. The isolates are grouped according to three regions of Thailand, Northern province in dark blue (*n* = 91), Northeastern province in light blue (*n* = 79) and Southern province in red (*n* = 9). The four Thai isolates of unknown origin are in black (*n* = 4). The percentage replicate trees in which the associated taxa clustered together in the bootstrap test (1000 replicates) more than 70% of the time (*n*≥70%) are indicated. The evolutionary distances were computed using the Maximum Composite Likelihood method and are in the units of the number of base substitutions per site.

A Principal Component Analysis (PCA) was used to assess the hierarchical structuring of the genetic population of *Cng* in Thailand. The genetic structure captured by the first two principal components was depicted by the individual genotypes (represented by dots) clustering into three groups and summarised by 95% ellipses. The typology of the individual allelic profiles revealed little differentiation between the 183 isolates from the three regions (figure 2). A maximum likelihood tree depicting the phylogenetic relationships within Thailand supported this genetic homogeneity, with all but the single isolates of STs 48 and 53 (CM21 and 50NC1 respectively; table 1) clustering together with high bootstrap support (bootstrap 100%; figure 3). Although identical to ST46 at six of the seven loci, 50NCI of ST53 was an outlier due to variations in its nucleotide sequence at *LAC1* (table 1). CM21's allelic profile, on the other hand, consisted of seven ATs which were not found in any other Thai isolate typed in this study.

### Population structure of the wider Asian population of *Cng*

Three isolates from the previously typed *Cng* population originated from HIV positive patients in Bangkok, Thailand [37], [55], and were of ST4 (th84, th206) and ST6 (th104; table S1). The STs of the newly typed Thai isolates consisted of a 12 nucleotide insertion at the *IGS1* locus, as well as a six and a three-nucleotide insertion at *SOD1*; these mutations were not found within the ATs of the previously typed Thai isolates (table S3). A further five isolates included in this study are of Asian origin: jp1086, jp1088 and J1 from Japan, and in2629 and in2632 from India (table S1). 25% of the variation between the Thai isolates typed in this study and the eight isolates of wider Asian origin was due to among population differences (data not shown). These eight previously typed isolates of Asian origin were combined with the 183 Thai isolates typed in this study to form the Asian population (*n* = 191) which was then compared to the remaining global isolates, also grouped according to geographic location: Africa (*n* = 44), North America (*n* = 19) and South America (*n* = 5).

### Genetic structure of the global population subdivided into geographically defined subpopulations

**Figure 4:**
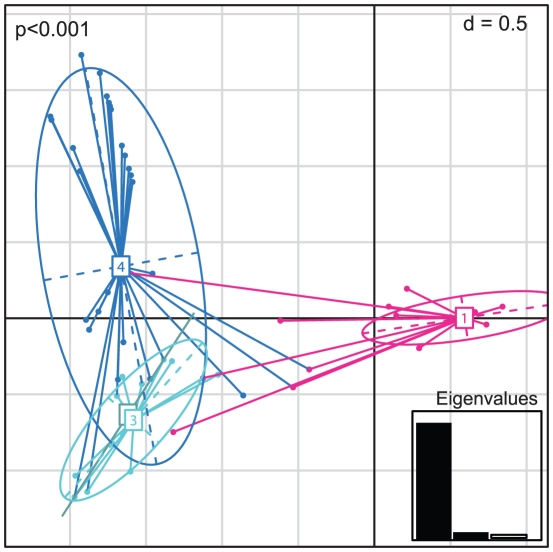
Principle Components Analysis of the allelic profiles of the global *Cng* genotypes analysed in this study. Individual genotypes (dots) are linked by coloured lines to form clusters which are summarised by coloured ellipses proportional in size to the number of isolates represented. The four groups are numbered and defined according continent: 1 = Asia (pink; *n* = 191), 2 = South America (grey; *n* = 5), 3 = North America (light blue; *n* = 19), 4 = Africa (dark blue; *n* = 44). *P -* value is shown and eigenvalues represented in the bar plot.

**Figure 5:**
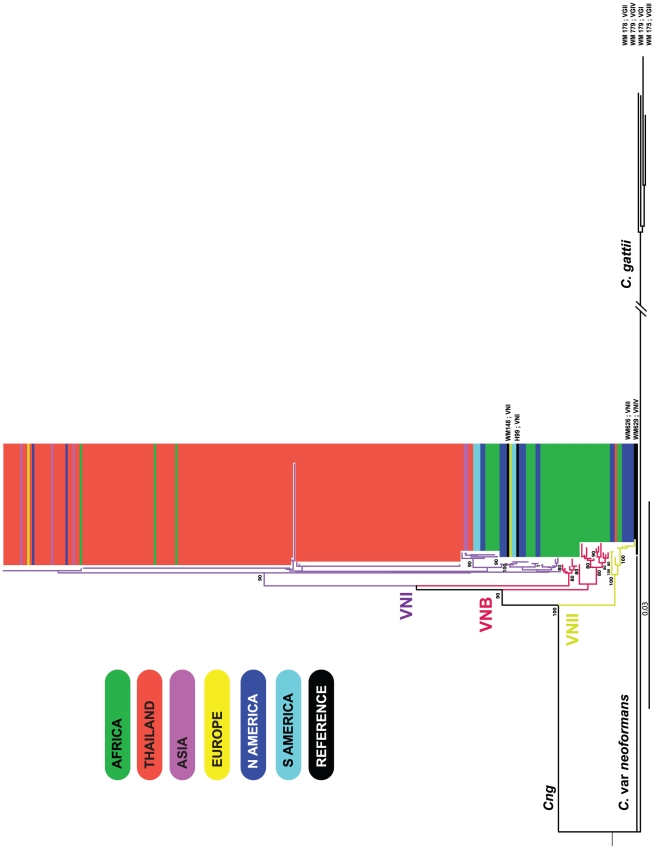
Neighbour-joining tree inferring the evolutionary relationships of the global *Cng* isolates included in this study (*n* = 261). The geographical origins of the isolates are represented by coloured rectangles: green  =  Africa (*n* = 44), red  =  Thailand (isolates typed in this study; *n* = 186), purple  =  remaining Asian isolates (*n* = 5), dark blue  =  North America (*n* = 19), light blue  =  South America (*n* = 5) and yellow  =  Europe (*n* = 2). Black rectangles represent reference strains of known VN molecular types that are detailed on the figure for VNI (WM148, H99; *n* = 232), VNII (WM626; *n* = 11) and VNB (*n* = 21). Reference strains of the *C. gattii* complex (molecular groups VGI – IV) are labelled and serve as an outgroup: WM179, WM178, WM175 and WM779. The percentage replicate trees in which the associated taxa clustered together in the bootstrap test (1000 replicates) are indicated if supported by significant bootstrap values (*n≥*80%). The evolutionary distances were computed using the Maximum Composite Likelihood method and are in the units of the number of base substitutions per site.

AMOVA attributed 52% of the variation in the global population of *Cng* to differences between the four geographically defined sub-populations (ΦPT = 0.52, *p* = 0.001; table 2). We excluded Europe due to a small sample size (*n* = 2). The first principal coordinate in the inter-class PCA for the global samples' allelic profiles distinguished the Asian population (pink ellipse, group 1) from the rest of the global population subsets (Africa, North and South America), *p*<0.001 (figure 4). A dendrogram inferring the relationships between all isolates delineated three major groups within the global population: VNI (*n* = 230; type isolates WM148, H99), VNII (*n* = 10; type isolates WM626) and VNB (*n* = 21; figure 5). Molecular group VNB was mostly found in Botswana, and consisted of three previously described sub-populations which were geographically and genetically isolated from lineages of *Cng* found elsewhere: VNB-A, VNB-B [56] and VNB-C [41]. Although confined to Botswana in this study, previous studies have reported the occurrence of VNB *Cn* Aα (also known as AFLP genotype 1A) infecting AIDS patients in Rwanda, the USA and Belgium, from the environment in Zaire and Australia and from both clinical and environmental samples in Brazil [13], [14], [22], South Africa and Columbia [57]. The origin of VNB has previously been hypothesised to be the result of hybridisation between VNI (serotype A, ALFP genotype 1) and VNIV (serotype D, AFLP genotype 2) [14], [37]. Eight of the ten African isolates of the rare mating type MAT**a** were from this group. All but one of the Thai isolates typed in this study clustered with the global VNI isolates, with the single isolate, CM21 of ST48 (table 1), falling within molecular group VNII along with reference strain WM626 (bootstrap value 100%; figure 5). Isolate CM21 being of a different VN group explains why it was an outlier in the maximum likelihood tree analysis of the phylogenetic relationships within the Thai STs (figure 3). In addition, isolate 50NCI, the second outlier of ST53, was found to correlate with the VNI group (WM148, H99), also supported by significant bootstrap value (*n* = 90%; figure 5). In accordance with our PCA, the global phylogenetic analysis showed the previously typed Thai isolates (th84, th206 and th104) grouped with the newly typed Thai isolates (bootstrap support = 70%), while the remaining Asian isolates (J1, jp1086, jp1088, in2629 and in2632) clustered with the Thai isolates within the VNI group (figure 5).

### Predominant clonality detected within the Asian *Cng* populations

The Index of Association (*I*_A_) [58] and 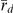 [59] were used to assess the overall association between alleles at the seven MLST loci, testing the null hypothesis of linkage equilibrium. A signature of clonal reproduction is the generation of non-random associations between loci, the amount of which can be estimated using linkage disequilibrium. Random association of alleles at the different loci was rejected for the sub-populations of isolates divided by geographic origin, with Africa having the lowest 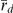 value (0.28, *p*<0.001; table 3). Clone-corrected data confirmed the predominance of clonal reproduction among the *Cng* samples. The proportion of phylogenetically compatible pairs of loci was used to test for linkage disequilibrium in the dataset, with the null hypothesis of free recombination being rejected if there were fewer than two locus pairs with all four allele combinations than expected under panmixis [60]. A significant percentage of phylogenetically compatible loci pairs was found for all geographically defined sub-populations (table 3), and the hypothesis of random mating rejected. The minimum number of recombination events (R_m_) [61] was estimated both within an individual locus and between loci (R_m_ and average R_m_ respectively; table 4) within described populations Africa, Asia and North America. Despite the main feature of the Asian population (*n* = 191) being strong clonality, some evidence for inter-locus recombination was detected (average R_m_ = 5; table 4). This was low in comparison with the African population, where an average R_m_ of 12 was observed. Africa also exhibited more intralocus recombination with 5/7 loci showing 1 or more inferred events, as opposed to 1/7 loci in Asia and North America. The locus with the highest inferrred intralocus R_m_ was *IGS1* for African, Asian and North American populations (table 4); a feature that is perhaps related to the multicopy nature of this locus. When analysed according to molecular group, recombination was detected within the VNI (*n* = 230) and VNB (*n* = 10) populations of the global isolates (R_m_ = 6 and 7, respectively; data not shown) and less so within the VNII population (*n* = 21, R_m_ = 1). The main feature of the Thai VNI *Cng* population is strong clonality, evidence of local clonal expansion within this geographical subset of the recombining global VNI population.

**Table 3:** Multilocus linkage disequilibrium analyses for samples of *Cn* var *grubii*.

Population#	Total sample			Population	Clone-corrected sample^a^		
	*I*_A_ b	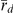 c	PcP^d^		*I*_A_	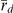	PcP
Africa(*n* = 44)	1.67***	0.28***	0.43***	Africa(*n* = 33)	1.25***	0.21***	0.43***
Asia(*n* = 191)	1.54***	0.30***	0.67***	Asia(*n* = 14)	1.11***	0.19***	0.67***
North America(*n* = 19)	3.45***	0.58***	1***	North America(*n* = 10)	2.13***	0.36***	1***
Global (*n* = 261)	3.18***	0.53***	0.19***	Global (*n* = 53)	1.53***	0.53***	0.38***

a excluding replicate haplotypes;

b index of association;

c scaled index of association (*I*_A_) by the number of loci (*m* – 1);

d percentage of phylogenetically compatible pairs (PcP) of loci.

***P<0.001.

# The South American and European populations were not individually analyzed due to their sample sizes being too small (*n* = 5 and 2, respectively), but were included in the global population (*n* = 261).

**Table 4:** Polymorphism summary and tests neutral evolution for groups of isolates of *Cn* var *grubii* according to geographic origin.

	Locus	pb^a^	S^b^	*h* c	*Hd* d	*π* e	*θ* f	D^g^	R_2_ h	Rm^i^ #
Africa (*n* = 44)	*CAP59*	501	11	10	0.82	0.004	0.005	-0.79_ns_	0.08_ns_	1
	*GPD1*	489	16	11	0.82	0.006	0.008	-0.55_ns_	0.09_ns_	0
	*IGS1*	704	22	12	0.83	0.006	0.007	-0.50_ns_	0.10_ns_	2
	*LAC1*	470	12	8	0.75	0.006	0.006	0.03_ns_	0.11_ns_	0
	*PLB1*	533	15	11	0.8	0.004	0.006	-1.09_ns_	0.07_ns_	1
	*SOD1*	524	24	10	0.64	0.011	0.011	0.30_ns_	0.12_ns_	1
	*URA5*	636	24	12	0.86	0.008	0.009	-0.43_ns_	0.10_ns_	1
			Average		0.79	0.007	0.007			12
Asia (*n* = 191)	*CAP59*	501	5	2	0.01	0.0001	0.002	-1.81*	0.07_ns_	0
	*GPD1*	489	6	3	0.28	0.0007	0.002	-1.40_ns_	0.06_ns_	0
	*IGS1*	707	11	3	0.06	0.0008	0.003	-1.71_ns_	0.03_ns_	0
	*LAC1*	474	61	6	0.64	0.0031	0.022	-2.62***	0.06_ns_	2
	*PLB1*	533	8	4	0.07	0.0003	0.003	-1.97*	0.05_ns_	0
	*SOD1*	526	11	2	0.01	0.0002	0.004	-2.25**	0.07_ns_	0
	*URA5*	637	10	4	0.33	0.0007	0.003	-1.78*	0.06_ns_	0
			Average		0.2	0.0001	0.005			5
North America (*n* = 19)	*CAP59*	501	8	5	0.78	0.006	0.005	1.39_ns_	0.20_ns_	0
	*GPD1*	489	7	5	0.76	0.006	0.004	1.28_ns_	0.20_ns_	0
	*IGS1*	708	16	6	0.77	0.008	0.006	1.09_ns_	0.18_ns_	2
	*LAC1*	471	9	5	0.8	0.008	0.005	1.77_ns_	0.22_ns_	0
	*PLB1*	533	9	5	0.81	0.007	0.005	1.65_ns_	0.21_ns_	0
	*SOD1*	526	12	4	0.57	0.01	0.007	1.80_ns_	0.21_ns_	0
	*URA5*	637	9	4	0.75	0.006	0.004	2.06*	0.23_ns_	0
			Average		0.75	0.007	0.005			4
South America (*n* = 5)	*CAP59*	501	1	2	0.6	0.001	0.001	1.22_ns_	0.3_ns_	0
	*GPD1*	489	0	1	0	0	0	ND	ND	ND
	*IGS1*	709	43	2	0.6	0.037	0.03	1.88*	0.3_ns_	0
	*LAC1*	470	2	2	0.6	0.003	0.002	1.46_ns_	0.3_ns_	0
	*PLB1*	533	1	2	0.6	0.001	0.001	1.22_ns_	0.3_ns_	0
	*SOD1*	527	0	1	0	0	0	ND	ND	ND
	*URA5*	637	1	2	0.6	0.001	0.001	1.22_ns_	0.3_ns_	0
			Average		0.4	0.006	0.005			0

a total number of sites in alignments, excluding indels and missing data;

b number of segregating sites;

c number of haplotypes;

d haplotypic diversity;

e average number of nucleotide differences per site;

f Watterson's estimate of the population scaled mutation rate, expressed per site [95];

g Tajima's D [62];

h Ramos-Onsins & Rozas' R_2_ [99];

I minimum number of recombination events [61];

# average Rm  =  Rm between all seven loci; ND not determined because of no polymorphism. ^ns^ non-significant (P>0.05),

*P<0.05, **P<0.01, **P<0.001.

### Subpopulations of the global *Cng* population are genetically divergent and differentiated

The average nucleotide diversity within geographically defined subpopulations was calculated at each locus and overall statistical tests included the number of segregating sites (*S*) and haplotypes (*h*), haplotypic diversity (*H_d_*), the number of nucleotide differences per site (*π*) and Watterson's estimate of the population scaled mutation rate (θ*).* Consistently higher average values of *H_d_*, *π* and of *θ* indicated higher levels of within-population variation among the African isolates than were observed in the Asian and South American populations. Similarly, the North American population's average values of *H_d_* (0.75) and θ (0.005) were lower than those of Africa (0.79 and 0.007, respectively; table 4).

Tajima's D tests the null hypothesis that populations are in mutation-drift equilibrium [62]. In the case of significant deviation from zero, the null hypothesis of neutral (random) evolution is rejected, a finding which can be due to the occurrence of natural selection or variable population dynamics. Significant departures from neutrality were detected at five of the seven loci of the Asian population (table 4), all of which had negative values. The remaining three global populations (Africa, North and South America) only had one or no significant departure from zero (table 4). Ramos-Onsins & Rozas' R_2_ test which is more powerful at detecting population growth [63] did not detect any deviation from random evolution among any of the populations (table 4).

The divergence among, and differentiation between, the four continental *Cng* populations were estimated using tests based on DNA sequences: the average nucleotide divergence between populations (D_xy_) [64], a weighted measure of the ratio of the average pair-wise differences within populations to the total average pairwise differences (K*_ST_)[65] and the nearest-neighbour statistic (S_nn_) [61], [66]. Low levels of nucleotide divergence were observed, with D_xy_ ranging from 0.3 and 0.7%, and no fixed differences found between the various continental populations at the seven loci (table 5A). The total number of shared polymorphisms among populations ranged from ten for Asia vs. South America, to 62 for Africa vs. North America, with locus *IGS1* contributing the most in each case (table 5A). The null hypothesis of no differentiation among populations of *Cng* was rejected for all populations paired with Asia due to significant K*_ST_ and S_nn_ values (table 5B). Africa and North America were also significantly differentiated, although considerably less so (K*_ST_ = 0.03, S_nn_ = 0.83), reflecting the high number of shared polymorphisms (table 5).

**Table 5:** (A) Divergence among the sub-populations of the global *Cng* isolates. (B) Differentiation between sub-populations of the global *Cng* isolates.

A.	Africa - Asia			Asia - North America			Asia - South America			Africa - North America			Africa - South America			North America - South America		
Locus	D_xy_ a	S_f_ b	Ss c	D_xy_	S_f_	Ss	D_xy_	S_f_	Ss	D_xy_	S_f_	Ss	D_xy_	S_f_	Ss	D_xy_	S_f_	Ss
CAP59	0.003	0	3	0.005	0	1	0.001	0	0	0.006	0	6	0.003	0	1	0.005	0	1
GPD1	0.007	0	5	0.005	0	5	0.003	0	0	0.007	0	6	0.005	0	0	0.004	0	0
IGS1	0.004	0	13	0.008	0	13	0.008	0	9	0.009	0	13	0.009	0	9	0.009	0	9
LAC1	0.006	0	4	0.008	0	2	0.004	0	0	0.008	0	9	0.005	0	2	0.007	0	2
PLB1	0.004	0	8	0.006	0	8	0.003	0	1	0.006	0	8	0.003	0	1	0.005	0	1
SOD1	0.008	0	11	0.008	0	12	0.000	0	0	0.013	0	11	0.008	0	0	0.008	0	0
URA5	0.007	0	9	0.006	0	8	0.002	0	0	0.001	0	9	0.006	0	1	0.005	0	1
**Average/total**	0.005	0	53	0.006	0	49	0.003	0	10	0.007	0	62	0.006	0	14	0.006	0	14
**B**.^d^^, ^ e			Africa		Asia		N. America		S. America									
	Africa				0.11***		0.03**		0.01ns									
	Asia		**0.95** ***				0.08***		0.04***									
	N. America		**0.83** ***		**0.96** ***				0.02ns									
	S. America		**0.86ns**		**0.99** ***		**0.74ns**											

The isolates are subdivided by continent: Africa (*n* = 44), Asia (*n* = 191), North and South America (*n* = 19 and 5, respectively).

a minimum estimate of the number of nucleotide differences per site between groups;

b number of fixed differences between groups;

c number of shared polymorphisms between groups.

d K_ST_* values are displayed above the diagonal and represent the weighted measure of the ratio of the average pair-wise differences within groups to the total average pair-wise differences.

e **S_nn_** values are displayed below the diagonal and in bold and represent the proportion of nearest neighbours in sequence space that are found in the same group.

Significance levels for K_ST_ and S_nn_ were assessed using permutation tests, with 1000 permutations:

ns  =  non-significant, **P<0.01, ***P<0.001.

Europe has been excluded as it contains only two isolates.

### Divergence time estimates and haplotype networks support a hypothesis of African ancestry for Asian *Cng* isolates

The time of divergence between the global subpopulations is defined as the mean time to most common recent ancestor (TMRCA) and was estimated using Bayesian markov-chain monte carlo (MCMC) methods in BEAST. Estimates obtained from runs of 10^7^ generations, according to three fixed substitution rates estimated for *Eurotiomycetes* [67] and assuming a relaxed log-normal clock, are shown in table 6. Two of the three mutation rates (0.9×10^-9^, 8.8×10^-9^) resulted in a TMRCA estimate whose upper and lower bounds span 5,000 years before present (y.b.p.). These values encompass the time of divergence proposed by the “Out of Africa” hypothesis for the global radiation of *Cng*. The highest effective sample size (ESS) was for an estimated rate of 0.9×10^-9^ substitutions per generation. We therefore estimated the mean TMRCA of the African and Asian population to be ≈ 6,921 y.b.p. (95% highest posterior density, HPD = 122–27,178) according to the best representative sample of the model used (XML file, dataset S1). Estimates of mean time to divergence for the two remaining populations were 5,090±1,419 y.b.p. (ESS = 42.09) for North America (*n* = 19) and 4,528±1,287 y.b.p. (ESS = 41.60) for South America (*n* = 5; data not shown).

**Table 6:** Bayesian estimates of time (in years) to the most recent common ancestor of *Cng* populations, according to geographic location, calculated under the assumption of three mutation rates and adopting the relaxed uncorrelated lognormal molecular clock model as implemented in BEAST v.1.4.1.

TMRCA	Mutation rates per site per year		
	0.9×10 ^-9^	8.8×10 ^-9^	16.7×10 ^-9^
Africa/Asia	6,921	60,572	1.05×10 ^6^
95% HPDI	(123 - 27,178)	(28 - 2.8×10^5^)	(3.8×10^5^-2.0×10^6^)
ESS	58.9	22.9	44.1
Global	7,103	60,739	1.05×10^6^
95% HPDI	(123 - 27,178)	(28-2.8×10 ^5^)	(3.8×10^5^-2.0×10 ^6^)
ESS	57.0	22.8	44.0

ESS  =  Effective sample size.

95% HPDI  = 95% highest posterior densities intervals.

**Figure 6:**
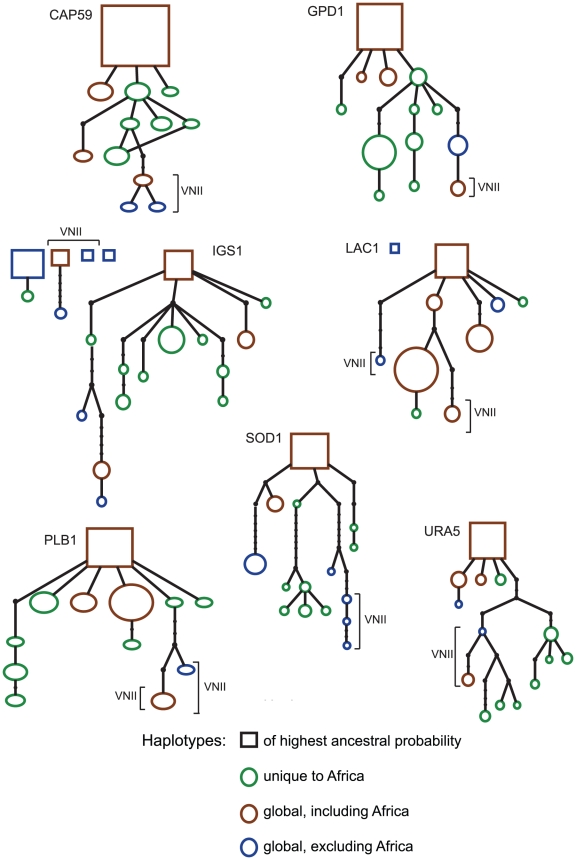
Haplotype networks of the 53 STs of the global *Cng* population at each of the seven loci. Sampled haplotypes are indicated by circles or rectangles colored according to the geographical region from which the sample was collected. STs unique to the African population are shown in green and consist only of clinical isolates. Haplotypes found both in Africa and elsewhere are in brown, while those not found in Africa are represented in blue. Rectangles depict the haplotype with the highest ancestral probability. Each branch indicates a single mutational difference and black dots on the lines are representative of the number of mutational steps required to generate allelic polymorphisms. Circle size is proportional to observed haplotype frequency.

To further explore the potential African ancestry of the *Cng* population, haplotype networks were constructed for each MLST locus (figure 6), as well as for the concatenated loci (figure S1). Sampled haplotypes are indicated by circles or rectangles colored according to the geographical region from which the sample was collected and proportional in size to observed haplotype frequency. Rectangles depict the haplotype with the highest ancestral probability and each branch indicates a single mutational difference. Internal nodes are representative of ancestral haplotypes, from which apical haplotypes evolved. The STs of non-African genotypes (shown in blue) were few and tended to be found at the apical (*ie.* derived) positions of the networks. The green circles, which represented STs of African origin only, were positioned throughout the networks but were only associated with clinical haplotypes. The combination of the seven networks pointed to an ancestral African population which had the highest variation in haplotype numbers and from which other global haplotypes were derived (figure S1).

### Associations between clinical variables and ST

There were no significant associations between the infecting ST and any of the reported baseline clinical variables indicative of disease progression. This lack of association is not surprising, as the genetically highly-related nature of these Thai genotypes is unlikely to lead to detectable variability in their clinical phenotype. The statistical power in this experiment was however sufficient to detect associations between clinical variables and disease progression as we found elevated baseline quantitative cryptococcal culture (range = 30 to 9,200,000) to be significantly associated with early death, with a 500,000 increment in CFU/ml/CSF resulting in a 30.6% increase in odds of death within ten weeks (*p* = 0.02). Similarly, altered mental status at presentation, defined by the presence of a decrease in Glasgow Coma scale or seizures, resulted in a 5.4 fold increased likelihood of death within 10 weeks (95% CI = 1.097 to 27.5; *p* = 0.02). These findings were consistent with previous observations made by Brouwer *et al*., 2004 [68]. The regression model best describing the prognostic factors of early death also included logarithmic interferon gamma (range = 0.32 to 2.23), which, when decreased by 0.1 in CSF, results in a 29% increase in odds of death within ten weeks (*p* = 0.02; table S4).

## Discussion

Affecting nearly 20% of HIV-AIDS patients nationwide, cryptococcosis is a leading AIDS-defining systemic infection in Thailand [38]. The high rates of mortality, re-admissions and relapses are attributed to a combination of factors that include high poverty rates resulting in few being able to afford timely antifungal treatment, the limitations of current antifungal drugs, the limited availability of highly active anti-retroviral therapy (HAART) and the trend of late presentation due to religious and cultural influences [69]. As the population of immunosuppressed individuals increases, the potential for the continued increase in the disease burden of AIDS–related meningitis cannot be ignored, particularly in the developing countries of Southeast Asia [8]. Continued global typing is the key to elucidating the population structure of *Cng* in order to understand the contribution of the pathogen's genotype to the epidemiology of this infection. Therefore, standardisation by ISHAM of the typing methodologies and nomenclature in the study of *Cng* has the potential to greatly facilitate global health efforts to increase our knowledge and surveillance of this pathogenic fungus [44].

We initially used MLST to describe the genetic structure of *Cng* in Thailand. All 183 isolates typed were of *Cng* (serotype A) and mating type α, consistent with previous reports that serotype A, mating type α, is the dominant cause of cryptococcosis among immunocompromised individuals, as well as predominating in the environment [1], [8], [15], [19], [43], [70], [71]. Similarly, all but one Thai isolate, CM21, were of molecular type VNI (figure 5), which is the most prevalent VN-type worldwide [15], [43], [72], as well as among Southeast Asian populations such as Thailand [16] and Malaysia [17]. MLST revealed ten sequence types (ST44 to 53), three of which accounted for 95% of the isolates typed. Two of these three STs (44 and 45) contained 81% of the 183 isolates (table 1) and differed at only four nucleotide positions within the *LAC1* locus. AMOVA showed that only 5% of the observed genetic variation across Thailand could be attributed to differences among the three regions (table 2), showing that *Cng* exhibits little spatial structure at this geographic scale. PCA (figure 2) and phylogenetic analyses (figure 3) support the conclusion that there is little geographical variation between the regional Thai *Cng* isolates that were typed in this study. This genetic pattern is consistent with that found in Cng isolates from five geographic locations within another Asian country, India [73].

Eight isolates within the previously typed *Cng* population [38] were of Asian origin (table S1). AMOVA revealed 25% of the molecular variance to be due to diversity between this wider Asian population (*n* = 8) and that of the Thai isolates typed in this study (*n* = 183). All the previously typed isolates clustered within groups of the Thai isolates with high bootstrap support, showing that they are highly related; for this reason they were subsequently combined to form the Asian population of *Cng* which was subsequently tested against the global sample of *Cng.*

Our analyses then focused on comparing the type and distribution of diversity between the different continental populations of *Cng*, and is the first time that a global analysis of the distribution of MLST polymorphisms has been undertaken for this pathogen. While sample sizes were low for two regions (Europe and South America), our power to detect differences between continents was satisfactory for the other sampled regions (North America, Africa and Asia). Our data and analyses clearly showed the following facets of *Cng*'s global population structure: 1. the fungus is widely clonally reproducing, 2. recombination, where observed, is geographically proscribed and 3. continental populations are differentiated and vary in their levels of diversity. Below, we discuss and integrate these findings.

Statistically significant tests of non-random association of alleles at the different loci (*I*_A_, 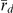 and PcP; table 3) demonstrated an overwhelmingly clonal population structure within the Asian population of *Cng*. Elsewhere, a similar pattern of clonality was seen for populations of *Cng* sampled from Africa and North America (clone corrected 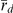 = 0.21 and 0.36 respectively, *p*<0.001). These results are consistent with previous studies showing that non-meiotic reproduction is the predominate mode of descent in *Cng* worldwide [12], [37], [41], [74], [75]. Having said this, recent investigation of the predominance of the α mating type in nature led to the finding that cryptococcal strains of the same mating type within serotypes A and D are capable of sexual reproduction in the form of haploid and monokaryotic fruiting, a process previously believed to be mitotic and asexual [76]. As there have been previous reports of recombination within predominantly clonal populations of *Cng* [56], [77], [78], including an environmental sample consisting of only MAT-α alleles in the Asian country of India [73], R_m_ was applied to the different sub-populations of *Cng* despite the strong clonal component detected. This technique detects the minimum number of recombination events that are necessary to explain the distribution of polymorphisms within and between loci. The test demonstrated a high degree of spatial variation in the rates of recombination globally (table 4). Importantly, the highest number of minimum recombination events was detected in the African population (Africa R_m_ = 12; Asia R_m_ = 5; North America R_m_ = 4) and the majority of the MLST loci in Africa showed evidence of intergenic recombination, in comparison with much lower levels detected elsewhere (Africa 5/7 loci; Asia 1/7 loci; North America 1/7 loci). These results are in keeping with studies reporting sexual propagation within both clinical [56] and environmental African isolates of *Cng* [79]. Furthermore, sub-divisions according to VN group showed the African VNB population (n = 21) to be highly recombining (Rm = 7) in comparison to the African VNI group (n = 21, Rm = 3; data not shown), likely due to the high frequency of the **a**-mating type detected in the former (table S1) [37].

Estimates of haplotypic diversity (*H_d_*), mutation rates (*θ*) and nucleotide differences (*π*) were consistently greater for Africa relative to populations in other continents (table 4). Africa exhibited the greatest number of haplotypes (Africa = 74> North America = 34> Asia = 24), and the Asian population exhibited the least amount of haplotypic diversity (Africa = 0.79> North America = 0.75> Asia = 0.20). Tajima's D is a statistical test that identifies loci that are evolving under non-random processes, such as selection or demographic expansion or contraction, and showed that 5/7 MLST loci in Asia were significantly non-neutral, compared to only 1/7 loci in North America and 0/7 in Africa. As the MLST loci used to type *Cng* are mostly in housekeeping genes [44], and therefore unlikely to be under strong selection, these differences in Tajima's D are most likely due to demographic effects such as population expansion following a population bottleneck. The possibility of neutrality could not be rejected within any of geographically defined population groups, according to the more powerful R_2_ statistical test (table 4), however the results qualitatively mirror those found for Tajima's D (table 4).

Global analyses of pairwise population combinations detected significant genetic differentiation between all *Cng* populations excepting the comparison between North and South America (table 5B), showing that the different continental populations of *Cng* are experiencing divergent evolutionary trajectories. The Asian population's comparatively low genetic diversity, high linkage disequilibrium, non-neutral evolution and lack of geographically defined structure are all consistent with a model of a rapid population expansion from a limited set of ancestors. This is supported by evidence of limited genetic variation within isolates from Northwest India, suggestive of recent origin and/or dispersal of Asian *Cng* isolates [73]. These findings contrast with the African population of *Cng*, which is characterised by high genetic diversity, balanced mating types and elevated recombination rates. This finding that the Asian isolates are genetically monomorphic in relation to African isolates led to our examining the potential of an ancestral African origin of *Cng* using coalescent analyses in BEAST. A substitution rate of 0.9×10^-9^ and a relaxed log-normal model estimated the time to ancestry of Africa/Asia to be at 6,920 y.b.p. with the 95% HDP levels of 123 – 27,178 (table 6). Ancestral estimations report a mean TMRCA of 5,090±1,419 y.b.p. for North America and 4,528±1,287 y.b.p for South America. However, these last two populations are considerably smaller (*n* = 19 and 5, respectively) leading to wide uncertainty. If a hypothesis of human trade-associated pigeon migration vectoring *Cng* is correct, one would expect Europe to follow Africa, but the current lack of data on *Cng* MLST genotypes in Europe means this cannot currently be tested. However, despite uncertainty in the exact order of the phylogenetic relationships, the 95% HPD estimates for ancestry between the Africa/Asia populations encompass the time frame of the domestication of the birds in Africa approximately 5,000 years ago prior to their introduction to Europe and subsequent distribution worldwide at two of the three substitution rates that we examined. Importantly, haplotype networks for each MLST network show that haplotypes unique to the African population occupy both internal and apical positions within the networks, whilst those unique to the global population are almost always at the derived positions at the network-tips. These data are persuasive evidence for the derivation of these lineages from an ancestral African population (figure 6, figure S1).

The invasion and expansion of two recombinant genotypes of *C. gattii* in the Pacific Northwest, and their differential virulence, has shown that genotypes of *Cryptococcus* can encode striking different clinical phenotypes [42]. We hypothesised that the bottlenecked diversity that we observe in our Thailand populations of *Cng* would translate into negligible difference in the progression of clinical disease between these highly-related ST's. The fact that one cohort of isolates collected from Sappasitprasong Hospital, Ubon Ratchathani, were highly characterised with respect to the progression of clinical disease following infection led us to test for a relationship between ST and the various clinical variables indicative of the progression of cryptococcosis in AIDS patients. While these sample sizes were sufficient to detect associations between clinical variables and disease progression, as has been previously described by Brouwer *et al* [68], we found no association between ST and disease progression. This is likely due to the fact that 95% of theses isolates were either of ST 44 or ST 45, which differ at only a single locus. As low genetic diversity appears to be the general condition in Asia *Cng*, the variation in clinical phenotype seen in this clinical sample appears overwhelmingly due to host effects as opposed to *Cng* genotype, whereas were we to look at an African cohort, effects owing to *Cng* genotype might be more apparent. A robust comparative analysis between African and Asian *Cng* using either experimental models or further clinical cohorts will be necessary to definitively answer this question.

Our study has shown that a genetically depauperate population of *Cng* infecting Thai HIV-AIDs patients shows many signatures of having been derived from a recombining African population across a timeframe that broadly encapsulates the anthropogenically driven globalisation of many major human infectious diseases. Further, our study has shown the gains that are associated with the collection of global MLST datasets, and sets the stage for integrating future MLST datasets, as well as utilising new deep-sequencing approaches to genotype whole *Cng* genomes in parallel. Further collaborative efforts by the *Cng* research community to integrate such genotyping approaches with spatial collections of isolates and clinical studies will lead to a better understanding of the evolution of this increasingly important, and understudied, emerging human pathogen.

## Materials and Methods

### Ethics statement

Ethical approval was required for the randomised control trial at Sappasitprasong Hospital, Ubon Ratchathani, the source of some isolates typed in this study. This was approved by the ethical and scientific review subcommittee of the Thai Ministry of Public Health and by the research ethics committee of St George's Hospital, London, UK, with written informed consent obtained for all 64 adults enrolled in this study.

### Isolates

The 183 Thailand isolates of *Cng* were acquired from three sources. Fifty-eight clinical isolates were collected during a randomised control trial at Sappasitprasong Hospital, Ubon Ratchathani, Northeast Thailand. This study aimed to compare the efficacy of four randomly assigned anti-fungal treatment combinations in the initial treatment of HIV-associated CM in an antiretroviral therapy (ART) naïve population, enrolling 64 adults with a first episode of cryptococcal meningitis [68]. A further 108 clinical isolates were obtained from a collection of cryptococcal samples managed by the CBS-KNAW Fungal Biodiversity Centre and originated from patients at various hospitals in three Thai regions: 76 in the North, 20 in the Northeast and 9 from the South. Three of these isolates were of unknown provenance. Of the total 173 clinical isolates, 154 (89%) were from HIV/AIDS patients with culture-proven *Cn* isolated from cerebrospinal fluid (*n* = 127), blood (*n* = 12) and broncho-alveolar lavage (*n* = 1). Three were from blood samples of HIV- negative CM patients. Eighteen cryptococcal isolates were provided by Dr. Pojana Sriburee, Chiang Mai University, ten of which were environmental and had been isolated from pigeon and dove guano [80]. One of the eight remaining isolates recovered from cryptococcosis patients was of Japanese origin, and was not considered as part of the Thai dataset (isolate J1; table S1). In total, these three collections yielded 183 isolates from 11 provinces in three regions of Thailand: North (*n* = 91), Northeast (*n* = 79) and South (*n* = 9), four unknown, 6% of which are environmental (table 1, figure 3).

These isolates were then compared to the global MLST dataset as compiled by A. Litvintseva [37], which consisted of 77 isolates whose genotypes and molecular groups had been previously determined by both amplified fragment length polymorphisms (AFLP) and MLST. All 261 *Cng* isolates, including the Japanese isolate J1, were grouped according to geographic origin: Asia (*n* = 191), Africa (*n* = 44), North America (*n* = 19), South America (*n* = 5), Europe (*n* = 2; table S1). As of the 2^nd^ of November, 2009, the MLST scheme contained 53 STs from 232 clinical, 20 environmental isolates and nine unknown of source, from 19 countries worldwide [37], [44] (table S1).

### Cultivation and DNA extraction

Isolates were cultured on pre-prepared malt extract agar (CM0059, Oxoid, Basingstoke, UK) and DNA extracted using the DNEASY Blood and Tissue Kit (Qiagen, Crawley, UK), then stored at 4°C prior to PCR-amplification. Samples of all cultures were subsequently cryopreserved in YPD (2.5 g Bacto yeast, 5 g Peptone, 5 g Dextrose and 250 ml dH_2_O) and 15% glycerol at -80°C.

### Mating-type and serotype analyses

The mating type of each of the isolates was determined by four different PCR amplification reactions. Primers specific to the MATα or MAT**a** allele of the STE20 locus for either serotype A or D isolates were used: primers JOHE7270 and JOHE7272 (aA), JOHE7273/JOHE7275 (aD), JOHE7264/JOHE7265 (αA) and JOHE7267/JOHE7268 (αD) [19], [22], [81]. PCR amplifications with a total volume of 25 µL contained 0.25 µL of 10 mM stock dNTPs, 0.25 µL Taq polymerase, 2.5 µL of buffer, 16.0 µl of sterilised distilled H_2_0, 1 µl of template DNA and 2.5 µL of each forward and reverse primer at a 10 µM final concentration.

### MLST determination

Each isolate was PCR-amplified in 50 µl reaction volumes for each of the seven MLST loci using the primers and protocols detailed in Meyer *et al.*, 2009 [45]. Each locus was subsequently sequenced using TaqFS (Big Dye V1.1) and an Applied Biosystems 3730XL sequencer (Warrington, UK) to determine the forward and reverse DNA sequences of all PCR products.

Sequences were manually edited using CodonCode Aligner (CodonCode Corporation, MA, USA), then aligned in MEGA 4.0 [82]. Alleles at each locus were assigned numbers (Allele Types; ATs) upon comparison with those identified in the global collection [37], resulting in a 7-digit allelic profile for each isolate. Each unique allelic profile was concatenated and assigned a Sequence Type (ST) according to the MLST scheme (http://cneoformans.mlst.net/). Novel STs identified within the Thai population were assigned as additional STs within the global MLST database. Data analyses were performed on both the Thai population of *Cn* typed in this study (*n* = 183), and on the complete global collection of strains (*n* = 261).

### Analysis of genetic structure based on allelic profiles

A hierarchical Analysis of Molecular Variance (AMOVA) was performed in GenAlEx 6.1 for Excel [83] in order to examine the distribution of genetic variation, and to determine the extent of connectivity among populations based on allelic profiles [84]. AMOVA is a statistical technique that estimates the extent of genetic differentiation between individuals and populations directly from molecular data. The technique treats the raw molecular data as a pairwise matrix of genetic distances between all the possible combinations of *Cng* isolates, with sub-matrices corresponding to the different hierarchical data-partitions (here, the genetic differences between *Cng* infecting different host individuals and geographical regions). The data is then analysed within a nested analysis of variance (ANOVA) framework. An F-statistic analogue of the genetic diversity among populations, ΦPT, and between pairs of groups (population pair wise ΦPT) is also reported [84], with significance estimated from 999 random permutations.

Patterns of allelic variability among the MLST genotypes of the Thai isolates typed in this study were investigated by Principle Component Analysis (PCA) using the Adegenet 1.1 package for statistical software R (version 2.6.1). This package is dedicated to the multivariate analysis of genetic markers, illustrating population stratification within a set of genotypes [85]. Diagrams obtained by PCA consist of dots, representing individual genotypes, clustered into groups. Isolates belonging to the same group are linked by matching coloured lines, labelled and summarised by 95% ellipses. Bar plots represent eigenvalues which describe the contributions of the principal coordinates to the genetic structure of the population depicted. Inter-class PCA was performed on the global population of *Cng*, also using Adegenet v1.1. This technique maximizes the variance between pre-defined groups as opposed to the total variance [86]. In order to assess the significance of this hierarchical data-structure, a Monte-Carlo procedure was applied.

### Phylogenetic analyses and molecular type determination

Phylogenetic neighbour-joining trees were inferred for each locus as well as concatenated sequences for both the Thai and the global populations, with evolutionary distances computed using the Maximum Composite Likelihood method in MEGA 4.0 [82], [87]. The percentage of replicate trees in which the associated taxa clustered together was estimated by the bootstrap test, inferred from 1000 replicates [88]. Molecular VN groupings of the Thai isolates were inferred through phylogenetic and comparative analyses with the global isolates (*n* = 77; table S1). The VN groupings of global isolates were previously determined using phylogenetic methods and non-hierarchical ordination analyses of both AFLP and MLST data [37]. We also included reference strains of known major molecular types of the *C. neoformans/C. gattii* species complex: WM148 (serotype A, VNI), WM626 (serotype A, VNII), WM629 (serotype D, VNIV), WM179 (serotype B, VGI), WM178 (serotype B, VGII), WM175 (serotype B, VGIII), WM779 (serotype C, VGIV) [15] and the genome-project strain H99 (serotype A, VNI) [89].

### Linkage disequilibrium and recombination

Evidence of linkage disequilibrium was tested for using two measures of index of association, I_A_ [58] and 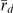 [59], [90], [91]. The significance of the pairwise statistics returned was determined by 1000 randomizations. In the instance of significant clonality or population substructure, both values are expected to be greater than zero, while freely recombining populations would return a score of zero. These tests were also performed on clone corrected samples as recombination may sometimes be masked by clonal reproduction. The proportion of phylogenetically compatible pairs of loci is also reported, with significance estimated with 1000 randomizations [92], [93].

The minimum number of recombination events (R_m_) was estimated based on the four-gametic test [61], both within individual locus and between loci within described subpopulations.

### Genetic variability and testing neutral expectations within individual populations

Comparative sequence analyses were performed in DnaSPv5 [94]. For each locus and each taxon, the number of segregating sites (*S*), haplotypes (*h*) and haplotypic diversity (*Hd*) [64] were calculated. The average number of nucleotide differences between pairs of sequences (*π*) [64] and the population scaled mutation rate estimated per site (*θ*) [95] are also reported. Tajima's D [62] and Ramos-Onsins and Rozas' R2 [63] were used to test for departures from the neutral model of molecular evolution, based on the site frequency spectrum. For both tests, significance was obtained from 10000 coalescent simulations.

### Genetic differentiation between populations

The average pair-wise number of nucleotide differences per site, D_xy_, was used to estimate divergence among population groups[64], while K*_ST_ (a weighted measure of the ratio of the average pair-wise differences within populations to the total average pairwise differences) [65] and S_nn_ (the proportion of nearest neighbours in sequence space found in the same population)[61], [66], were used to assess differentiation between the populations. These statistics were also calculated in DNASPv5, with significance levels assessed by 1000 permutations.

### Estimates of times of divergence and haplotype networks

A Bayesian Markov Chain Monte Carlo (MCMC) method, implemented in the program BEAST version 1.5.3 [96], was used to estimate the time of divergence between the geographically-defined populations of the global sample of *Cng*, defined as the time to the most recent common ancestor (TMRCA). Sequence indels greater than a single nucleotide long were treated as single evolutionary events in the dataset, and a second partition reflecting these indels created in Beauti v1.5.3 (XML file, dataset S1). The Hasegawa-Kishino-Yano (HKY) model of sequence evolution was assumed, and a relaxed, uncorrelated lognormal molecular clock model applied due to initial runs revealing standard deviation estimates of branch rates to be greater than the mean rate (σ>1), indicative of substantial rate heterogeneity among data lineages [96]. Simulations were run for 10^7^ with an initial burn-in of 10%. Parameters were logged every 1000 steps over the course of the run. We applied fixed substitution rates, allowing us to convert parameter estimates to calendar years. The rates used were 0.9×10^-9^, 8.8×10^-9^ and 16.7×10^-9^ mutations per site per year. These are the lower, mean, and upper bounds of a range of substitution rates estimated for *Eurotiomycetes*, based on a calibration date of 400 Myr [67]. Credibility intervals were obtained using 95% highest posterior density (HPD) intervals, the shortest segment that includes 95% of the probability density of the parameter, and the effective sample sizes (ESS) for each parameter, depicted using Tracer v1.5.

Haplotype networks were also created for the STs of the global *Cng* population at each MLST locus. The inference of phylogenetic relationships among them using statistical parsimony was performed using the program TCS v1.21 [97].

### Clinical data and analysis

Clinical data indicative of the progression of cryptococcal infection was available for 58 of the 174 Thai clinical isolates typed in this study. These data were collected previously during a randomized control trial at Sappasitprasong Hospital, Ubon Ratchathani, Thailand. The study aimed to compare the efficacy of four randomly assigned anti-fungal treatment combinations in the initial treatment of HIV-associated CM [68]. Data available included baseline measurements of cerebrospinal fluid (CSF) opening pressure (cm), quantitative cryptococcal CSF culture (CFU/ml CSF), and logarithmic interferon gamma levels. Fungicidal activity was defined by the reduction in CSF cryptococcal colony-forming units (CFU) from quantitative CSF cultures measured at three intervals over the two weeks of treatment. Cerebral dysfunction upon presentation and time to death were also reported [68].

We investigated potential associations between ST and baseline continuous variables using both ANOVA and multivariate ANOVA (MANOVA), with Fisher's exact test being applied to categorical variables. Logistic regression was used to determine factors associated with death by 10 weeks. All analyses were performed using statistical software package R (version 2.6.1).

### MLST website eBURST tool

eBURST, a program available at http://eburst.mlst.net/, infers patterns of evolutionary descent among clusters of related genotypes from MLST data. eBURST utilises the MLST site's geographical mapping of MLST data sets (figure S2) to subdivide the STs into related groups of or clonal complexes, as well as to identify the founding genotype (ST) of each group [98].

### Accession numbers

All genotypes mentioned within this manuscript are publically available on the MLST database at http://cneoformans.mlst.net/, numbered according to ST as detailed in table S1.

## Supporting Information

Table S1 The allelic profiles of the 261 global *Cng* isolates typed at the seven loci as determined by the ISHAM MLST included in this study.
(0.55 MB DOC)

Table S2 Diversity indices of the Thai *Cng* population.
(0.03 MB DOC)

Table S3 Distribution of nucleotide polymorphisms and insertions within MLST genes IGS1 and SOD1 *Cng* allele types according to the respective position at which it was observed.
(0.19 MB DOC)

Table S4 Logistic regression model best describing the prognostic factors of early death (by 10 weeks) among the Thai HIV/AIDS patients.
(0.03 MB DOC)

Figure S1 Haplotype networks of the 53 concatenated STs of the global *Cng* population. Sampled haplotypes are indicated by circles or rectangles colored according to the geographical region from which the sample was collected. STs unique to the African population are shown in green and consist only of clinical isolates. Haplotypes found both in Africa and elsewhere are in brown, while those not found in Africa are represented in blue. Rectangles depict the haplotype with the highest ancestral probability. Each branch indicates a single mutational difference and black dots on the lines are representative of the number of mutational steps required to generate allelic polymorphisms. Circle size is proportional to observed haplotype frequency.
(0.17 MB PDF)

Figure S2 MLST map of the current global *Cng* isolates. This screenshot of the current distribution of Cng isolates worldwide (*n* = 261) depicted by the MLST website represents the mapping tool utilised in comparative eBURST analysis of Cng populations.
(0.33 MB PNG)

Dataset S1 XML file of the current global population of *Cng* assuming a relaxed log-normal clock and a fixed substitution rate of 0.9 x 10-9 per generation.
(1.09 MB XML)
