# Supplementary material for: Low Diversity Cryptococcus neoformans Variety grubii Multilocus Sequence Types from Thailand Are Consistent with an Ancestral African Origin
Source: PLoS Pathog. 2011 Apr 28;7(4):e1001343. doi: 10.1371/journal.ppat.1001343 (PMC3089418; doi:10.1371/journal.ppat.1001343)
Supplement: Table S1 — The allelic profiles of the 261 global Cng isolates typed at the seven loci as determined by the ISHAM MLST included in this study. (0.55 MB DOC) [file ppat.1001343.s001.doc]

| **Molecular type** | **Name** | **Mating type** | **CAP59 allele** | **GPD1 allele** | **IGS allele** | **LAC1 allele** | **PLB1 allele** | **SOD1 allele** | **URA5 allele** | **ST** | **Strain origin (if known)** |
| --- | --- | --- | --- | --- | --- | --- | --- | --- | --- | --- | --- |
| VNI | CN5010 | α | 1 | 1 | 19 | 3 | 2 | 13 | 5 | 44 | Chiang Rai, Thailand, blood |
|  | CN4998 | α | 1 | 1 | 19 | 3 | 2 | 13 | 5 | 44 | Chiang Mai, Thailand, CSF |
|  | CN4995 | α | 1 | 1 | 19 | 3 | 2 | 13 | 5 | 44 | Chiang Mai, Thailand, CSF |
|  | CN4989 | α | 1 | 1 | 19 | 3 | 2 | 13 | 5 | 44 | Chiang Mai, Thailand, CSF |
|  | CN4988 | α | 1 | 1 | 19 | 3 | 2 | 13 | 5 | 44 | Chiang Mai, Thailand, CSF |
|  | CN4987 | α | 1 | 1 | 19 | 3 | 2 | 13 | 5 | 44 | Chiang Mai, Thailand, CSF |
|  | CN4964 | α | 1 | 1 | 19 | 3 | 2 | 13 | 5 | 44 | Chiang Mai, Thailand, CSF |
|  | CN4947 | α | 1 | 1 | 19 | 3 | 2 | 13 | 5 | 44 | Chiang Rai, Thailand, CSF |
|  | CN4945 | α | 1 | 1 | 19 | 3 | 2 | 13 | 5 | 44 | Chiang Rai, Thailand, CSF |
|  | CN4944 | α | 1 | 1 | 19 | 3 | 2 | 13 | 5 | 44 | Chiang Mai, Thailand, CSF |
|  | CN4943 | α | 1 | 1 | 19 | 3 | 2 | 13 | 5 | 44 | Chiang Rai, Thailand |
|  | CN4942 | α | 1 | 1 | 19 | 3 | 2 | 13 | 5 | 44 | Lampang, Thailand, CSF |
|  | CN4941 | α | 1 | 1 | 19 | 3 | 2 | 13 | 5 | 44 | Thailand, CSF |
|  | CN4940 | α | 1 | 1 | 19 | 3 | 2 | 13 | 5 | 44 | Thailand, CSF |
|  | CN4926 | α | 1 | 1 | 19 | 3 | 2 | 13 | 5 | 44 | Chiang Rai, Thailand, CSF |
|  | CN4919 | α | 1 | 1 | 19 | 3 | 2 | 13 | 5 | 44 | Chiang Rai, Thailand, CSF |
|  | CN4918 | α | 1 | 1 | 19 | 3 | 2 | 13 | 5 | 44 | Chiang Rai, Thailand, CSF |
|  | CN4917 | α | 1 | 1 | 19 | 3 | 2 | 13 | 5 | 44 | Chiang Rai, Thailand, CSF |
|  | CN4903 | α | 1 | 1 | 19 | 3 | 2 | 13 | 5 | 44 | Chiang Rai, Thailand, CSF |
|  | CN4901 | α | 1 | 1 | 19 | 3 | 2 | 13 | 5 | 44 | Chiang Mai, Thailand, CSF |
|  | CN49005 | α | 1 | 1 | 19 | 3 | 2 | 13 | 5 | 44 | Chiang Mai, Thailand |
|  | 4-187 | α | 1 | 1 | 19 | 3 | 2 | 13 | 5 | 44 | Khon Kaen, Thailand, clinical |
|  | 269 | α | 1 | 1 | 19 | 3 | 2 | 13 | 5 | 44 | Khon Kaen, Thailand, clinical |
|  | 4-315 | α | 1 | 1 | 19 | 3 | 2 | 13 | 5 | 44 | Khon Kaen, Thailand, clinical |
|  | 1-587 | α | 1 | 1 | 19 | 3 | 2 | 13 | 5 | 44 | Khon Kaen, Thailand, clinical |
|  | 1219 | α | 1 | 1 | 19 | 3 | 2 | 13 | 5 | 44 | Khon Kaen, Thailand, clinical |
|  | 4_83 | α | 1 | 1 | 19 | 3 | 2 | 13 | 5 | 44 | Khon Kaen, Thailand, clinical |
|  | 1-588 | α | 1 | 1 | 19 | 3 | 2 | 13 | 5 | 44 | Khon Kaen, Thailand, clinical |
|  | 4-202 | α | 1 | 1 | 19 | 3 | 2 | 13 | 5 | 44 | Khon Kaen, Thailand, clinical |
|  | 1-846 | α | 1 | 1 | 19 | 3 | 2 | 13 | 5 | 44 | Khon Kaen, Thailand, clinical |
|  | 2551-07 | α | 1 | 1 | 19 | 3 | 2 | 13 | 5 | 44 | Songkhla, Thailand, CSF |
|  | 2550 II-07 | α | 1 | 1 | 19 | 3 | 2 | 13 | 5 | 44 | Songkhla, Thailand, blood |
|  | 2461-07 | α | 1 | 1 | 19 | 3 | 2 | 13 | 5 | 44 | Songkhla, Thailand, CSF |
|  | CM 1 | α | 1 | 1 | 19 | 3 | 2 | 13 | 5 | 44 | Ubon Ratchathani, Thailand, CSF |
|  | CM 6 | α | 1 | 1 | 19 | 3 | 2 | 13 | 5 | 44 | Ubon Ratchathani, Thailand, CSF |
|  | CM 7 | α | 1 | 1 | 19 | 3 | 2 | 13 | 5 | 44 | Ubon Ratchathani, Thailand, CSF |
|  | CM 8 | α | 1 | 1 | 19 | 3 | 2 | 13 | 5 | 44 | Ubon Ratchathani, Thailand, CSF |
|  | CM 12 | α | 1 | 1 | 19 | 3 | 2 | 13 | 5 | 44 | Ubon Ratchathani, Thailand, CSF |
|  | CM 13 | α | 1 | 1 | 19 | 3 | 2 | 13 | 5 | 44 | Ubon Ratchathani, Thailand, CSF |
|  | CM 17 | α | 1 | 1 | 19 | 3 | 2 | 13 | 5 | 44 | Ubon Ratchathani, Thailand, CSF |
|  | CM 18 | α | 1 | 1 | 19 | 3 | 2 | 13 | 5 | 44 | Ubon Ratchathani, Thailand, CSF |
|  | CM 22 | α | 1 | 1 | 19 | 3 | 2 | 13 | 5 | 44 | Ubon Ratchathani, Thailand, CSF |
|  | CM 23 | α | 1 | 1 | 19 | 3 | 2 | 13 | 5 | 44 | Ubon Ratchathani, Thailand, CSF |
|  | CM 25 | α | 1 | 1 | 19 | 3 | 2 | 13 | 5 | 44 | Ubon Ratchathani, Thailand, CSF |
|  | CM 26 | α | 1 | 1 | 19 | 3 | 2 | 13 | 5 | 44 | Ubon Ratchathani, Thailand, CSF |
|  | CM 33 | α | 1 | 1 | 19 | 3 | 2 | 13 | 5 | 44 | Ubon Ratchathani, Thailand, CSF |
|  | CM 37 | α | 1 | 1 | 19 | 3 | 2 | 13 | 5 | 44 | Ubon Ratchathani, Thailand, CSF |
|  | CM 38 | α | 1 | 1 | 19 | 3 | 2 | 13 | 5 | 44 | Ubon Ratchathani, Thailand, CSF |
|  | CM 39 | α | 1 | 1 | 19 | 3 | 2 | 13 | 5 | 44 | Ubon Ratchathani, Thailand, CSF |
|  | CM 40 | α | 1 | 1 | 19 | 3 | 2 | 13 | 5 | 44 | Ubon Ratchathani, Thailand, CSF |
|  | CM 41 | α | 1 | 1 | 19 | 3 | 2 | 13 | 5 | 44 | Ubon Ratchathani, Thailand, CSF |
|  | CM42 | α | 1 | 1 | 19 | 3 | 2 | 13 | 5 | 44 | Ubon Ratchathani, Thailand, CSF |
|  | CM 43 | α | 1 | 1 | 19 | 3 | 2 | 13 | 5 | 44 | Ubon Ratchathani, Thailand, CSF |
|  | CM 44 | α | 1 | 1 | 19 | 3 | 2 | 13 | 5 | 44 | Ubon Ratchathani, Thailand, CSF |
|  | CM 46 | α | 1 | 1 | 19 | 3 | 2 | 13 | 5 | 44 | Ubon Ratchathani, Thailand, CSF |
|  | CM 47 | α | 1 | 1 | 19 | 3 | 2 | 13 | 5 | 44 | Ubon Ratchathani, Thailand, CSF |
|  | CM 48 | α | 1 | 1 | 19 | 3 | 2 | 13 | 5 | 44 | Ubon Ratchathani, Thailand, CSF |
|  | CM 49 | α | 1 | 1 | 19 | 3 | 2 | 13 | 5 | 44 | Ubon Ratchathani, Thailand, CSF |
|  | CM 51 | α | 1 | 1 | 19 | 3 | 2 | 13 | 5 | 44 | Ubon Ratchathani, Thailand, CSF |
|  | CM 55 | α | 1 | 1 | 19 | 3 | 2 | 13 | 5 | 44 | Ubon Ratchathani, Thailand, CSF |
|  | CM 56 | α | 1 | 1 | 19 | 3 | 2 | 13 | 5 | 44 | Ubon Ratchathani, Thailand, CSF |
|  | CM 57 | α | 1 | 1 | 19 | 3 | 2 | 13 | 5 | 44 | Ubon Ratchathani, Thailand, CSF |
|  | CM 58 | α | 1 | 1 | 19 | 3 | 2 | 13 | 5 | 44 | Ubon Ratchathani, Thailand, CSF |
|  | CM 59 | α | 1 | 1 | 19 | 3 | 2 | 13 | 5 | 44 | Ubon Ratchathani, Thailand, CSF |
|  | CM 61 | α | 1 | 1 | 19 | 3 | 2 | 13 | 5 | 44 | Ubon Ratchathani, Thailand, CSF |
|  | CM 63 | α | 1 | 1 | 19 | 3 | 2 | 13 | 5 | 44 | Ubon Ratchathani, Thailand, CSF |
|  | K 2 | α | 1 | 1 | 19 | 3 | 2 | 13 | 5 | 44 | Khon Kaen, Thailand, cryptococcosis patient |
|  | Pg 1 | α | 1 | 1 | 19 | 3 | 2 | 13 | 5 | 44 | Chiang Mai, Thailand, pigeon dropping |
|  | D 6 | α | 1 | 1 | 19 | 3 | 2 | 13 | 5 | 44 | Chiang Mai, Thailand, dove dropping |
|  | D 1 | α | 1 | 1 | 19 | 3 | 2 | 13 | 5 | 44 | Chiang Mai, Thailand, dove dropping |
|  | CN5019 | α | 1 | 1 | 19 | 4 | 2 | 13 | 5 | 45 | Chiang Rai, Thailand, blood |
|  | CN5017 | α | 1 | 1 | 19 | 4 | 2 | 13 | 5 | 45 | Chiang Rai, Thailand, CSF |
|  | CN5014 | α | 1 | 1 | 19 | 4 | 2 | 13 | 5 | 45 | Chiang Rai, Thailand, blood |
|  | CN5013 | α | 1 | 1 | 19 | 4 | 2 | 13 | 5 | 45 | Chiang Rai, Thailand, CSF |
|  | CN5011 | α | 1 | 1 | 19 | 4 | 2 | 13 | 5 | 45 | Thailand, clinical |
|  | CN5009 | α | 1 | 1 | 19 | 4 | 2 | 13 | 5 | 45 | Chiang Rai, Thailand, blood |
|  | CN5005 | α | 1 | 1 | 19 | 4 | 2 | 13 | 5 | 45 | Chiang Rai, Thailand, blood |
|  | CN5003 | α | 1 | 1 | 19 | 4 | 2 | 13 | 5 | 45 | Chiang Rai, Thailand, blood |
|  | CN5002 | α | 1 | 1 | 19 | 4 | 2 | 13 | 5 | 45 | Chiang Rai, Thailand, blood |
| **Molecular type** | **Name** | **Mating type** | **CAP59 allele** | **GPD1 allele** | **IGS allele** | **LAC1 allele** | **PLB1 allele** | **SOD1 allele** | **URA5 allele** | **ST** | **Strain origin (if known)** |
|  | CN5001 | α | 1 | 1 | 19 | 4 | 2 | 13 | 5 | 45 | Chiang Rai, Thailand, CSF |
|  | CN4970 | α | 1 | 1 | 19 | 4 | 2 | 13 | 5 | 45 | Chiang Mai, Thailand, CSF |
|  | CN4968 | α | 1 | 1 | 19 | 4 | 2 | 13 | 5 | 45 | Chiang Mai, Thailand, CSF |
|  | CN4957 | α | 1 | 1 | 19 | 4 | 2 | 13 | 5 | 45 | Chiang Rai, Thailand, CSF |
|  | CN4956 | α | 1 | 1 | 19 | 4 | 2 | 13 | 5 | 45 | Chiang Rai, Thailand, CSF |
|  | CN4955 | α | 1 | 1 | 19 | 4 | 2 | 13 | 5 | 45 | Thailand, BAL |
|  | CN4954 | α | 1 | 1 | 19 | 4 | 2 | 13 | 5 | 45 | Lampang, Thailand, CSF |
|  | CN4952 | α | 1 | 1 | 19 | 4 | 2 | 13 | 5 | 45 | Tak, Thailand, CSF |
|  | CN4950 | α | 1 | 1 | 19 | 4 | 2 | 13 | 5 | 45 | Lampoon, Thailand, CSF |
|  | CN4949 | α | 1 | 1 | 19 | 4 | 2 | 13 | 5 | 45 | Lampoon, Thailand, CSF |
|  | CN4938 | α | 1 | 1 | 19 | 4 | 2 | 13 | 5 | 45 | Chiang Mai, Thailand, CSF |
|  | CN4937 | α | 1 | 1 | 19 | 4 | 2 | 13 | 5 | 45 | Chiang Mai, Thailand, CSF |
|  | CN4936 | α | 1 | 1 | 19 | 4 | 2 | 13 | 5 | 45 | Chiang Mai, Thailand, CSF |
|  | CN4934 | α | 1 | 1 | 19 | 4 | 2 | 13 | 5 | 45 | Chiang Mai, Thailand, CSF |
|  | CN4933 | α | 1 | 1 | 19 | 4 | 2 | 13 | 5 | 45 | Chiang Mai, Thailand, CSF |
|  | CN4932 | α | 1 | 1 | 19 | 4 | 2 | 13 | 5 | 45 | Chiang Mai, Thailand, CSF |
|  | CN4931 | α | 1 | 1 | 19 | 4 | 2 | 13 | 5 | 45 | Chiang Mai, Thailand, CSF |
|  | CN4927 | α | 1 | 1 | 19 | 4 | 2 | 13 | 5 | 45 | Chiang Mai, Thailand, CSF |
|  | CN4915 | α | 1 | 1 | 19 | 4 | 2 | 13 | 5 | 45 | Chiang Mai, Thailand, CSF |
|  | CN4914 | α | 1 | 1 | 19 | 4 | 2 | 13 | 5 | 45 | Chiang Mai, Thailand, CSF |
|  | CN4909 | α | 1 | 1 | 19 | 4 | 2 | 13 | 5 | 45 | Chiang Mai, Thailand, CSF |
|  | CN4907 | α | 1 | 1 | 19 | 4 | 2 | 13 | 5 | 45 | Chiang Mai, Thailand, CSF |
|  | CN4905 | α | 1 | 1 | 19 | 4 | 2 | 13 | 5 | 45 | Chiang Mai, Thailand, CSF |
|  | CN4904 | α | 1 | 1 | 19 | 4 | 2 | 13 | 5 | 45 | Chiang Mai, Thailand, CSF |
|  | CN4902 | α | 1 | 1 | 19 | 4 | 2 | 13 | 5 | 45 | Chiang Mai, Thailand, CSF |
|  | CN49008 | α | 1 | 1 | 19 | 4 | 2 | 13 | 5 | 45 | Chiang Mai, Thailand, CSF |
|  | 4-319 | α | 1 | 1 | 19 | 4 | 2 | 13 | 5 | 45 | Khon Kaen, Thailand, clinical |
|  | 50NC2 | α | 1 | 1 | 19 | 4 | 2 | 13 | 5 | 45 | Nan, Thailand, clinical |
|  | 50NC5 | α | 1 | 1 | 19 | 4 | 2 | 13 | 5 | 45 | Nan, Thailand, clinical |
|  | 11112 | α | 1 | 1 | 19 | 4 | 2 | 13 | 5 | 45 | Khon Kaen, Thailand, clinical |
|  | 11109 | α | 1 | 1 | 19 | 4 | 2 | 13 | 5 | 45 | Khon Kaen, Thailand, clinical |
|  | 4-231 | α | 1 | 1 | 19 | 4 | 2 | 13 | 5 | 45 | Khon Kaen, Thailand, clinical |
|  | P6 | α | 1 | 1 | 19 | 4 | 2 | 13 | 5 | 45 | Chiang Mai, Thailand, clinical |
|  | 4-253 | α | 1 | 1 | 19 | 4 | 2 | 13 | 5 | 45 | Khon Kaen, Thailand, clinical |
|  | 4-381 | α | 1 | 1 | 19 | 4 | 2 | 13 | 5 | 45 | Khon Kaen, Thailand, clinical |
|  | 20662-07 | α | 1 | 1 | 19 | 4 | 2 | 13 | 5 | 45 | Songkhla, Thailand, blood |
|  | 28170-07 | α | 1 | 1 | 19 | 4 | 2 | 13 | 5 | 45 | Songkhla, Thailand, CSF |
|  | 1111I-08 | α | 1 | 1 | 19 | 4 | 2 | 13 | 5 | 45 | Pattani, Thailand, blood/HIV- |
|  | 2895I-08 | α | 1 | 1 | 19 | 4 | 2 | 13 | 5 | 45 | Pattani, Thailand, blood/HIV- |
|  | 4500-07 | α | 1 | 1 | 19 | 4 | 2 | 13 | 5 | 45 | Pattani, Thailand, blood |
|  | CM 2 | α | 1 | 1 | 19 | 4 | 2 | 13 | 5 | 45 | Ubon Ratchathani, Thailand, CSF |
|  | CM 3 | α | 1 | 1 | 19 | 4 | 2 | 13 | 5 | 45 | Ubon Ratchathani, Thailand, CSF |
|  | CM 4 | α | 1 | 1 | 19 | 4 | 2 | 13 | 5 | 45 | Ubon Ratchathani, Thailand, CSF |
|  | CM 5 | α | 1 | 1 | 19 | 4 | 2 | 13 | 5 | 45 | Ubon Ratchathani, Thailand, CSF |
|  | CM 10 | α | 1 | 1 | 19 | 4 | 2 | 13 | 5 | 45 | Ubon Ratchathani, Thailand, CSF |
|  | CM 14 | α | 1 | 1 | 19 | 4 | 2 | 13 | 5 | 45 | Ubon Ratchathani, Thailand, CSF |
|  | CM 11 | α | 1 | 1 | 19 | 4 | 2 | 13 | 5 | 45 | Ubon Ratchathani, Thailand, CSF |
|  | CM 15 | α | 1 | 1 | 19 | 4 | 2 | 13 | 5 | 45 | Ubon Ratchathani, Thailand, CSF |
|  | CM16 | α | 1 | 1 | 19 | 4 | 2 | 13 | 5 | 45 | Ubon Ratchathani, Thailand, CSF |
|  | CM 20 | α | 1 | 1 | 19 | 4 | 2 | 13 | 5 | 45 | Ubon Ratchathani, Thailand, CSF |
|  | CM 24 | α | 1 | 1 | 19 | 4 | 2 | 13 | 5 | 45 | Ubon Ratchathani, Thailand, CSF |
|  | CM 27 | α | 1 | 1 | 19 | 4 | 2 | 13 | 5 | 45 | Ubon Ratchathani, Thailand, CSF |
|  | CM 28 | α | 1 | 1 | 19 | 4 | 2 | 13 | 5 | 45 | Ubon Ratchathani, Thailand, CSF |
|  | CM 29 | α | 1 | 1 | 19 | 4 | 2 | 13 | 5 | 45 | Ubon Ratchathani, Thailand, CSF |
|  | CM 32 | α | 1 | 1 | 19 | 4 | 2 | 13 | 5 | 45 | Ubon Ratchathani, Thailand, CSF |
|  | CM 34 | α | 1 | 1 | 19 | 4 | 2 | 13 | 5 | 45 | Ubon Ratchathani, Thailand, CSF |
|  | CM 36 | α | 1 | 1 | 19 | 4 | 2 | 13 | 5 | 45 | Ubon Ratchathani, Thailand, CSF |
|  | CM 45 | α | 1 | 1 | 19 | 4 | 2 | 13 | 5 | 45 | Ubon Ratchathani, Thailand, CSF |
|  | CM 50 | α | 1 | 1 | 19 | 4 | 2 | 13 | 5 | 45 | Ubon Ratchathani, Thailand, CSF |
|  | CM 52 | α | 1 | 1 | 19 | 4 | 2 | 13 | 5 | 45 | Ubon Ratchathani, Thailand, CSF |
|  | CM 60 | α | 1 | 1 | 19 | 4 | 2 | 13 | 5 | 45 | Ubon Ratchathani, Thailand, CSF |
|  | CM 64 | α | 1 | 1 | 19 | 4 | 2 | 13 | 5 | 45 | Ubon Ratchathani, Thailand, CSF |
|  | Pt 9 | α | 1 | 1 | 19 | 4 | 2 | 13 | 5 | 45 | Chiang Mai, Thailand, cryptococcosis patient |
|  | Pt 3 | α | 1 | 1 | 19 | 4 | 2 | 13 | 5 | 45 | Chiang Mai, Thailand, cryptococcosis patient |
|  | Pt 1 | α | 1 | 1 | 19 | 4 | 2 | 13 | 5 | 45 | Chiang Mai, Thailand, cryptococcosis patient |
|  | D 2 | α | 1 | 1 | 19 | 4 | 2 | 13 | 5 | 45 | Chiang Mai, Thailand, dove dropping |
|  | D 3 | α | 1 | 1 | 19 | 4 | 2 | 13 | 5 | 45 | Chiang Mai, Thailand, dove dropping |
|  | Pg 2 | α | 1 | 1 | 19 | 4 | 2 | 13 | 5 | 45 | Chiang Mai, Thailand, pigeon dropping |
|  | Pg 26 | α | 1 | 1 | 19 | 4 | 2 | 13 | 5 | 45 | Chiang Mai, Thailand, pigeon dropping |
|  | CN49004 | α | 1 | 3 | 19 | 5 | 2 | 13 | 1 | 46 | Chiang Mai, Thailand, CSF |
|  | CN48 | α | 1 | 3 | 19 | 5 | 2 | 13 | 1 | 46 | Khon Kaen, Thailand, clinical |
|  | 1-488 | α | 1 | 3 | 19 | 5 | 2 | 13 | 1 | 46 | Khon Kaen, Thailand, clinical |
|  | 1-489 | α | 1 | 3 | 19 | 5 | 2 | 13 | 1 | 46 | Khon Kaen, Thailand, clinical |
|  | CM 30 | α | 1 | 3 | 19 | 5 | 2 | 13 | 1 | 46 | Ubon Ratchathani, Thailand, CSF |
|  | Pt 12 | α | 1 | 3 | 19 | 5 | 2 | 13 | 1 | 46 | Chiang Mai, Thailand, cryptococcosis patient |
|  | D 5 | α | 1 | 3 | 19 | 5 | 2 | 13 | 1 | 46 | Chiang Mai, Thailand, dove dropping |
|  | Pg 37 | α | 1 | 3 | 19 | 5 | 2 | 13 | 1 | 46 | Chiang Mai, Thailand, pigeon dropping |
|  | CN5015 | α | 1 | 3 | 19 | 5 | 2 | 13 | 1 | 46 | Chiang Rai, Thailand, CSF |
|  | CN5018 | α | 1 | 3 | 19 | 5 | 2 | 13 | 1 | 46 | Chiang Rai, Thailand, blood |
| **Molecular type** | **Name** | **Mating type** | **CAP59 allele** | **GPD1 allele** | **IGS allele** | **LAC1 allele** | **PLB1 allele** | **SOD1 allele** | **URA5 allele** | **ST** | **Strain origin (if known)** |
|  | CN5012 | α | 1 | 3 | 19 | 5 | 2 | 13 | 1 | 46 | Chiang Rai, Thailand, CSF |
|  | CN5008 | α | 1 | 3 | 19 | 5 | 2 | 13 | 1 | 46 | Chiang Rai, Thailand, CSF |
|  | CN4993 | α | 1 | 3 | 19 | 5 | 2 | 13 | 1 | 46 | Chiang Mai, Thailand, CSF |
|  | CN4983 | α | 1 | 3 | 19 | 5 | 2 | 13 | 1 | 46 | Chiang Mai, Thailand, CSF |
|  | CN4980 | α | 1 | 3 | 19 | 5 | 2 | 13 | 1 | 46 | Chiang Mai, Thailand, CSF |
|  | CN4977 | α | 1 | 3 | 19 | 5 | 2 | 13 | 1 | 46 | Chiang Mai, Thailand, CSF |
|  | CN4967 | α | 1 | 3 | 19 | 5 | 2 | 13 | 1 | 46 | Chiang Mai, Thailand, CSF |
|  | CN4960 | α | 1 | 3 | 19 | 5 | 2 | 13 | 1 | 46 | Chiang Rai, Thailand, CSF |
|  | CN4948 | α | 1 | 3 | 19 | 5 | 2 | 13 | 1 | 46 | Chiang Mai, Thailand, CSF |
|  | CN4946 | α | 1 | 3 | 19 | 5 | 2 | 13 | 1 | 46 | Chiang Mai, Thailand, CSF |
|  | CN4924 | α | 1 | 3 | 19 | 5 | 2 | 13 | 1 | 46 | Chiang Mai, Thailand, CSF |
|  | CN4921 | α | 1 | 3 | 19 | 5 | 2 | 13 | 1 | 46 | Mae Hong Son, Thailand, CSF |
|  | CN4920 | α | 1 | 3 | 19 | 5 | 2 | 13 | 1 | 46 | Chiang Mai, Thailand, CSF |
|  | CN4916 | α | 1 | 3 | 19 | 5 | 2 | 13 | 1 | 46 | Chiang Mai, Thailand, CSF |
|  | CN4906 | α | 1 | 3 | 19 | 5 | 2 | 13 | 1 | 46 | Chiang Mai, Thailand, CSF |
|  | J 1 | α | 1 | 3 | 19 | 5 | 2 | 13 | 1 | 46 | Japan, cryptococcosis patient |
|  | CN49006 | α | 1 | 3 | 19 | 5 | 2 | 13 | 1 | 46 | Chiang Mai, Thailand, CSF |
|  | 50NC1 | α | 1 | 3 | 19 | 10 | 2 | 13 | 1 | 53 | Nan, Thailand, clinical |
|  | Pt 5 | α | 1 | 1 | 19 | 5 | 2 | 13 | 1 | 51 | Chiang Mai, Thailand, cryptococcosis patient |
|  | CN5007 | α | 1 | 1 | 20 | 3 | 4 | 13 | 1 | 47 | Chiang Rai, Thailand, CSF |
|  | 1291-09 | α | 1 | 1 | 20 | 3 | 4 | 13 | 1 | 47 | Pattani, Thailand, blood/HIV- |
|  | CM 35 | α | 1 | 1 | 20 | 3 | 4 | 13 | 1 | 47 | Ubon Ratchathani, Thailand, CSF |
|  | K 45 | α | 1 | 1 | 19 | 3 | 4 | 13 | 5 | 50 | Khon Kaen, Thailand, cryptococcosis patient |
|  | 4_9 | α | 1 | 1 | 19 | 9 | 2 | 13 | 5 | 52 | Khon Kaen, Thailand, clinical |
|  | D 9 | α | 1 | 1 | 19 | 4 | 2 | 13 | 14 | 49 | Chiang Mai, Thailand, dove dropping |
|  | th84 | α | 1 | 1 | 1 | 4 | 2 | 1 | 5 | 4 | Thailand, blood/HIV+ |
|  | th206 | α | 1 | 1 | 1 | 4 | 2 | 1 | 5 | 4 | Thailand, blood/HIV+ |
|  | th104 | α | 1 | 1 | 1 | 3 | 2 | 1 | 5 | 6 | Thailand, blood/HIV+ |
|  | jp1086 | α | 1 | 3 | 1 | 5 | 2 | 1 | 1 | 5 | Japan, human lung |
|  | jp1088 | α | 1 | 3 | 1 | 5 | 2 | 1 | 1 | 5 | Japan, human lung |
|  | in2629 | α | 1 | 1 | 10 | 3 | 1 | 1 | 1 | 38 | India, CSF/AIDS |
|  | in2632 | α | 1 | 1 | 10 | 3 | 1 | 1 | 1 | 38 | India, CSF/AIDS |
|  | A4 34-6 | α | 1 | 1 | 11 | 3 | 4 | 1 | 1 | 39 | North Carolina, USA, pigeon excreta |
|  | it743 | α | 1 | 3 | 1 | 5 | 2 | 1 | 1 | 5 | Italy |
|  | c8 | α | 1 | 3 | 1 | 5 | 2 | 1 | 1 | 5 | North Carolina, USA, CSF/HIV+ |
|  | A5 35-17 | α | 1 | 3 | 1 | 5 | 2 | 1 | 1 | 5 | North Carolina, USA, pigeon excreta |
|  | A2 28-23 | α | 1 | 2 | 6 | 3 | 2 | 1 | 1 | 15 | North Carolina, USA, pigeon excreta |
|  | A2 102-5 | α | 1 | 2 | 6 | 3 | 2 | 1 | 1 | 15 | Texas, USA, pigeon excreta |
|  | c26 | α | 7 | 1 | 1 | 2 | 1 | 1 | 2 | 23 | North Carolina, USA, blood/HIV+ |
|  | A3 1-1 | α | 7 | 1 | 1 | 2 | 1 | 1 | 2 | 23 | North Carolina, USA, pigeon excreta |
|  | A3 38-20 | α | 7 | 1 | 1 | 1 | 1 | 1 | 1 | 1 | North Carolina, USA, pigeon excreta |
|  | arg1373 | α | 7 | 1 | 1 | 1 | 1 | 1 | 2 | 2 | Argentina |
|  | arg1366 | α | 7 | 1 | 1 | 1 | 1 | 1 | 2 | 2 | Argentina |
|  | c23 | α | 7 | 1 | 1 | 1 | 1 | 1 | 2 | 2 | North Carolina, USA , BAL/HIV- |
|  | A1 35-8 | α | 7 | 1 | 1 | 1 | 1 | 1 | 2 | 2 | North Carolina, USA, pigeon excreta |
|  | A1 | α | 7 | 1 | 1 | 1 | 1 | 1 | 2 | 2 | North Carolina, USA, pigeon excreta |
|  | ma120 | α | 7 | 1 | 1 | 1 | 1 | 1 | 2 | 2 | Malawi, blood/AIDS patient |
|  | Tn470 | α | 7 | 1 | 1 | 1 | 1 | 1 | 2 | 2 | Tanzania, blood/HIV+ |
|  | JH125.91 | a | 1 | 1 | 1 | 3 | 4 | 1 | 1 | 3 | Tanzania |
|  | Tn148 | α | 1 | 1 | 1 | 4 | 2 | 1 | 5 | 4 | Tanzania, blood/HIV+ |
|  | ug2463 | α | 1 | 1 | 1 | 3 | 2 | 1 | 5 | 6 | Uganda, CSF/HIV+ |
|  | bt68 | α | 1 | 5 | 1 | 1 | 4 | 1 | 1 | 13 | Botswana, CSF/AIDS |
|  | bt130 | a | 1 | 5 | 1 | 1 | 4 | 1 | 1 | 13 | Botswana, CSF/AIDS |
|  | bt150 | α | 7 | 5 | 1 | 1 | 3 | 4 | 9 | 21 | Botswana, CSF/AIDS |
|  | bt100 | α | 7 | 5 | 1 | 1 | 5 | 2 | 9 | 24 | Botswana, CSF/AIDS |
|  | bt104 | α | 1 | 6 | 1 | 1 | 4 | 1 | 1 | 22 | Botswana, CSF/AIDS |
|  | bt15 | α | 1 | 6 | 1 | 1 | 4 | 1 | 1 | 22 | Botswana, CSF/AIDS |
|  | ug2467 | α | 7 | 1 | 1 | 2 | 1 | 1 | 2 | 23 | Uganda, CSF/HIV+ |
|  | ug2459 | α | 7 | 1 | 1 | 2 | 1 | 1 | 2 | 23 | Uganda, CSF/HIV+ |
|  | A4 1-12 | α | 1 | 1 | 1 | 1 | 1 | 1 | 2 | 37 | France, environmental |
|  | bt134 | α | 1 | 3 | 1 | 5 | 4 | 1 | 1 | 25 | Botswana, CSF/AIDS |
|  | mal 9 | α | 2 | 1 | 10 | 3 | 2 | 1 | 5 | 30 | Malawi, blood/AIDS |
|  | ug2471 | α | 1 | 1 | 10 | 3 | 2 | 1 | 1 | 31 | Uganda, CSF/HIV+ |
|  | br2362 | α | 1 | 1 | 10 | 3 | 4 | 1 | 1 | 32 | Brazil |
|  | br794 | α | 1 | 1 | 10 | 3 | 4 | 1 | 1 | 32 | Brazil |
|  | br795 | α | 1 | 1 | 10 | 3 | 4 | 1 | 1 | 32 | Brazil |
|  | ug2458 | α | 1 | 1 | 10 | 3 | 4 | 1 | 1 | 32 | Uganda, CSF/HIV+ |
|  | Tn10 | α | 1 | 1 | 10 | 3 | 4 | 1 | 1 | 32 | Tanzania, blood/HIV+ |
|  | za1346 | α | 1 | 1 | 10 | 3 | 4 | 1 | 1 | 32 | Zaire, CSF/HIV+ |
|  | c27 | α | 1 | 1 | 10 | 3 | 4 | 1 | 1 | 32 | North Carolina, USA, CSF/cancer |
|  | za1345 | α | 1 | 1 | 10 | 3 | 4 | 1 | 1 | 32 | Zaire |
|  | bt121 | α | 1 | 5 | 18 | 1 | 4 | 1 | 1 | 34 | Botswana, CSF/AIDS |
|  | bt9 | α | 1 | 7 | 3 | 1 | 4 | 1 | 9 | 36 | Botswana, CSF/AIDS |
| VNII | CM 21 | α | 2 | 10 | 21 | 6 | 11 | 14 | 4 | 48 | Ubon Ratchathani, Thailand, CSF |
|  | ug2462 | α | 2 | 9 | 14 | 8 | 11 | 12 | 4 | 40 | Uganda, CSF/HIV+ |
|  | ug2472 | α | 2 | 9 | 14 | 8 | 11 | 12 | 4 | 40 | Uganda, CSF/HIV+ |
|  | c45 | α | 10 | 9 | 14 | 8 | 11 | 12 | 4 | 41 | North Carolina, USA, sputum/HIV- |
|  | c2 | α | 8 | 10 | 15 | 8 | 12 | 3 | 11 | 42 | North Carolina, USA, BAL/HIV- |
|  | c44 | α | 8 | 10 | 15 | 8 | 12 | 3 | 11 | 42 | North Carolina, USA, CSF/HIV- |
| **Molecular type** | **Name** | **Mating type** | **CAP59 allele** | **GPD1 allele** | **IGS allele** | **LAC1 allele** | **PLB1 allele** | **SOD1 allele** | **URA5 allele** | **ST** | **Strain origin (if known)** |
|  | c12 | α | 2 | 9 | 14 | 8 | 11 | 11 | 4 | 43 | North Carolina, USA, lung/HIV- |
|  | JH8-1 | α | 2 | 9 | 14 | 8 | 11 | 11 | 4 | 43 | North Carolina, USA |
|  | c16 | α | 2 | 9 | 14 | 8 | 11 | 11 | 4 | 43 | North Carolina, USA, sputum/HIV- |
|  | A7 35-23 | α | 2 | 9 | 14 | 8 | 11 | 11 | 4 | 43 | North Carolina, USA, pigeon excreta |
| VNB | bt31 | α | 4 | 11 | 9 | 6 | 7 | 1 | 12 | 8 | Botswana, CSF/AIDS |
|  | bt109 | α | 4 | 11 | 7 | 6 | 4 | 1 | 3 | 9 | Botswana, CSF/AIDS |
|  | bt33 | α | 4 | 11 | 7 | 6 | 4 | 1 | 3 | 9 | Botswana, CSF/AIDS |
|  | bt206 | a | 4 | 11 | 2 | 6 | 4 | 7 | 3 | 16 | Botswana, CSF/AIDS |
|  | bt89 | α | 4 | 11 | 2 | 6 | 4 | 7 | 3 | 16 | Botswana, CSF/AIDS |
|  | bt35 | α | 4 | 11 | 2 | 6 | 6 | 6 | 3 | 18 | Botswana, CSF/AIDS |
|  | bt27 | α | 5 | 11 | 2 | 6 | 4 | 1 | 6 | 35 | Botswana, CSF/AIDS |
|  | bt84 | α | 5 | 11 | 16 | 6 | 6 | 5 | 3 | 17 | Botswana, CSF/AIDS |
|  | bt76 | α | 5 | 11 | 7 | 6 | 6 | 7 | 10 | 11 | Botswana, CSF/AIDS |
|  | bt88 | a | 5 | 11 | 7 | 6 | 6 | 1 | 10 | 28 | Botswana, CSF/AIDS |
|  | bt65 | a | 9 | 11 | 2 | 6 | 4 | 1 | 3 | 10 | Botswana, CSF/AIDS |
|  | bt1 | α | 9 | 11 | 2 | 6 | 10 | 7 | 6 | 7 | Botswana, CSF/AIDS |
|  | bt46 | α | 11 | 11 | 2 | 6 | 6 | 7 | 6 | 19 | Botswana, CSF/AIDS |
|  | bt24 | a | 6 | 8 | 13 | 6 | 9 | 8 | 8 | 14 | Botswana, CSF/AIDS |
|  | bt63 | a | 6 | 8 | 13 | 6 | 8 | 8 | 7 | 20 | Botswana, CSF/AIDS |
|  | bt204 | a | 6 | 13 | 12 | 6 | 9 | 8 | 13 | 29 | Botswana, CSF/AIDS |
|  | bt85 | **a** | 12 | 8 | 17 | 6 | 9 | 9 | 8 | 27 | Botswana, CSF/AIDS |
|  | bt60 | α | 4 | 8 | 2 | 7 | 9 | 9 | 7 | 33 | Botswana, CSF/AIDS |
|  | bt131 | **a** | 3 | 12 | 2 | 6 | 6 | 6 | 8 | 12 | Botswana, CSF/AIDS |
|  | bt125 | α | 5 | 4 | 8 | 3 | 10 | 10 | 1 | 26 | Botswana, CSF/AIDS |
|  |  |  |  |  |  |  |  |  |  |  |  |

CSF = cerebrospinal fluid, BAL = broncho-alveolar lavage, HIV = Human Immunodeficiency Virus
