# Supplementary material for: Low Diversity Cryptococcus neoformans Variety grubii Multilocus Sequence Types from Thailand Are Consistent with an Ancestral African Origin
Source: PLoS Pathog. 2011 Apr 28;7(4):e1001343. doi: 10.1371/journal.ppat.1001343 (PMC3089418; doi:10.1371/journal.ppat.1001343)
Supplement: Table S2 — Diversity indices of the Thai Cng population. (0.03 MB DOC) [file ppat.1001343.s002.doc]

| Thailand | Locus | *pb* ^a^ | *S*^b^ | *h* ^c^ | *Hd* ^d^ | *π* ^e^ | *θ* ^f^ |
| --- | --- | --- | --- | --- | --- | --- | --- |
| (*n* =183) | CAP59 | 501 | 5 | 2 | 0.01 | 0.0001 | 0.002 |
|  | GPD1 | 489 | 6 | 3 | 0.27 | 0.0006 | 0.002 |
|  | IGS1 | 721 | 12 | 3 | 0.04 | 0.0006 | 0.003 |
|  | LAC1 | 467 | 61 | 6 | 0.64 | 0.0032 | 0.023 |
|  | PLB1 | 533 | 7 | 3 | 0.05 | 0.0002 | 0.002 |
|  | SOD1 | 529 | 11 | 2 | 0.01 | 0.0002 | 0.004 |
|  | URA5 | 637 | 10 | 4 | 0.31 | 0.0006 | 0.003 |
|  |  |  |  | Average | 0.19 | 0.0008 | 0.005 |

^a^ total number of sites in alignments, excluding indels and missing data; ^b^ number of segregating sites; ^c^ number of haplotypes; ^d^ haplotypic diversity; ^e^ average number of nucleotide differences per site; ^f^ Watterson’s estimate of the population scaled mutation rate, expressed per site [95].
