# Supplementary material for: Low Diversity Cryptococcus neoformans Variety grubii Multilocus Sequence Types from Thailand Are Consistent with an Ancestral African Origin
Source: PLoS Pathog. 2011 Apr 28;7(4):e1001343. doi: 10.1371/journal.ppat.1001343 (PMC3089418; doi:10.1371/journal.ppat.1001343)
Supplement: Table S3 — Distribution of nucleotide polymorphisms and insertions within MLST genes IGS1 and SOD1 Cng allele types according to the respective position at which it was observed. (0.19 MB DOC) [file ppat.1001343.s003.doc]

| **SOD1** |  |  |  |  |  | 1 | 1 | 1 | 1 | 1 | 1 | 2 | 2 | 3 | 3 | 3 | 3 | 3 | 3 | 3 | 3 | 3 | 3 | 3 | 3 | 4 | 4 | 4 | 4 | 5 | 5 | 5 | 5 | 5 | 5 |
| --- | --- | --- | --- | --- | --- | --- | --- | --- | --- | --- | --- | --- | --- | --- | --- | --- | --- | --- | --- | --- | --- | --- | --- | --- | --- | --- | --- | --- | --- | --- | --- | --- | --- | --- | --- |
|  | 4 | 4 | 7 | 7 | 7 | 3 | 3 | 6 | 6 | 7 | 9 | 7 | 9 | 1 | 3 | 3 | 3 | 3 | 4 | 4 | 4 | 4 | 8 | 9 | 9 | 3 | 3 | 5 | 7 | 1 | 1 | 1 | 1 | 1 | 3 |
|  | 2 | 8 | 0 | 1 | 2 | 6 | 8 | 2 | 9 | 4 | 9 | 9 | 9 | 8 | 6 | 7 | 8 | 9 | 0 | 1 | 2 | 4 | 8 | 8 | 9 | 1 | 6 | 2 | 8 | 0 | 1 | 2 | 6 | 8 | 4 |
|  |  |  |  |  |  |  |  |  |  |  |  |  |  |  |  |  |  |  |  |  |  |  |  |  |  |  |  |  |  |  |  |  |  |  |  |
| AT1 | A | T | A | A | A | C | T | T | A | G | C | C | G | A | T | - | - | - | - | - | - | A | G | C | G | G | C | G | A | - | - | - | A | G | C |
| AT2 | . | . | . | . | . | . | . | . | . | . | . | . | . | . | . | - | - | - | - | - | - | . | . | T | . | . | . | . | . | - | - | - | C | . | . |
| AT3 | . | . | . | . | . | . | . | G | G | . | T | T | . | . | C | - | - | - | - | - | - | . | . | . | . | A | T | . | . | - | - | - | . | . | T |
| AT4 | . | . | . | . | . | . | . | . | . | . | . | . | . | . | . | - | - | - | - | - | - | . | . | T | . | . | . | . | . | - | - | - | . | . | . |
| AT5 | . | . | . | . | . | . | . | . | . | . | . | . | . | . | . | - | - | - | - | - | - | . | . | . | . | . | T | . | G | - | - | - | . | . | . |
| AT6 | C | . | . | . | . | G | A | . | . | A | T | T | A | G | . | - | - | - | - | - | - | G | . | . | . | . | T | A | G | - | - | - | . | . | . |
| AT7 | . | . | . | . | . | G | A | . | . | A | T | T | A | G | . | - | - | - | - | - | - | G | . | . | . | . | T | A | G | - | - | - | . | A | . |
| AT8 | . | . | . | . | . | G | A | . | . | A | T | T | A | G | . | - | - | - | - | - | - | G | . | . | . | . | T | A | G | - | - | - | . | . | . |
| AT9 | . | . | . | . | . | G | A | . | . | A | T | T | A | G | . | - | - | - | - | - | - | G | . | . | A | . | T | A | G | - | - | - | . | . | . |
| AT10 | . | . | . | . | . | G | A | . | . | A | T | T | A | G | . | - | - | - | - | - | - | G | A | . | . | . | T | . | G | - | - | - | . | . | . |
| AT11 | . | A | C | G | T | . | . | G | . | . | T | T | . | G | C | - | - | - | - | - | - | . | . | . | . | A | T | . | . | - | - | - | . | . | T |
| AT12 | . | A | C | G | . | . | . | G | . | . | T | T | . | G | C | - | - | - | - | - | - | . | . | . | . | A | T | . | . | - | - | - | . | . | T |
| **AT13** | . | . | . | . | . | . | . | . | . | . | . | . | . | . | . | **A** | **T** | **T** | **T** | **C** | **G** | . | . | . | . | . | . | . | . | **G** | **G** | **A** | . | . | . |
| **AT14** | . | A | C | G | T | . | . | G | . | . | T | T | . | G | C | - | - | - | - | - | - | . | . | . | . | A | T | . | . | **G** | **G** | **A** | . | . | T |
|  |  |  |  |  |  |  |  |  |  |  |  |  |  |  |  |  |  |  |  |  |  |  |  |  |  |  |  |  |  |  |  |  |  |  |  |

| **IGS1** |  |  |  | 1 | 1 | 2 | 2 | 2 | 3 | 3 | 3 | 3 | 3 | 3 | 3 | 3 | 3 | 3 | 3 | 3 | 3 | 3 | 3 | 3 | 3 | 4 | 4 | 4 | 4 | 4 | 4 | 4 | 4 | 5 | 5 | 5 | 5 | 5 | 5 | 6 | 6 | 6 |
| --- | --- | --- | --- | --- | --- | --- | --- | --- | --- | --- | --- | --- | --- | --- | --- | --- | --- | --- | --- | --- | --- | --- | --- | --- | --- | --- | --- | --- | --- | --- | --- | --- | --- | --- | --- | --- | --- | --- | --- | --- | --- | --- |
|  | 3 | 4 | 6 | 6 | 7 | 4 | 4 | 6 | 0 | 2 | 2 | 4 | 6 | 8 | 8 | 9 | 9 | 9 | 9 | 9 | 9 | 9 | 9 | 9 | 9 | 0 | 2 | 3 | 5 | 7 | 7 | 9 | 9 | 3 | 3 | 4 | 6 | 6 | 7 | 3 | 4 | 5 |
|  | 6 | 2 | 8 | 0 | 1 | 3 | 4 | 5 | 4 | 3 | 9 | 2 | 1 | 8 | 9 | 0 | 1 | 2 | 3 | 4 | 5 | 6 | 7 | 8 | 9 | 1 | 2 | 2 | 6 | 0 | 7 | 3 | 7 | 5 | 9 | 2 | 6 | 8 | 1 | 5 | 2 | 6 |
|  |  |  |  |  |  |  |  |  |  |  |  |  |  |  |  |  |  |  |  |  |  |  |  |  |  |  |  |  |  |  |  |  |  |  |  |  |  |  |  |  |  |  |
| AT1 | T | T | A | A | T | C | C | G | A | G | G | T | A | - | - | - | - | - | - | - | - | - | - | - | - | C | G | C | T | T | A | A | G | G | G | A | G | G | G | A | T | A |
| AT2 | . | . | . | . | . | . | . | . | . | . | . | . | . | - | - | - | - | - | - | - | - | - | - | - | - | . | A | . | . | . | . | . | . | . | . | . | . | . | . | . | . | . |
| AT3 | . | . | . | . | . | . | . | . | . | . | . | . | . | - | - | - | - | - | - | - | - | - | - | - | - | . | . | . | . | . | G | . | . | . | . | . | . | . | . | . | . | . |
| AT4* | G | . | . | . | . | . | . | . | . | . | . | . | . | - | - | - | - | - | - | - | - | - | - | - | - | . | . | . | . | . | . | . | . | . | . | . | . | . | . | **-** | . | . |
| AT5* | . | . | . | . | . | . | . | . | . | . | . | . | G | - | - | - | - | - | - | - | - | - | - | - | - | . | A | . | A | . | . | . | . | . | . | . | . | . | . | . | . | . |
| AT6 | . | . | T | . | . | . | . | . | . | . | A | C | . | - | - | - | - | - | - | - | - | - | - | - | - | T | . | . | . | . | . | . | . | . | . | . | . | . | . | . | . | . |
| AT7 | . | C | . | . | . | . | . | . | . | . | . | . | . | - | - | - | - | - | - | - | - | - | - | - | - | . | A | . | . | C | . | . | . | . | . | G | . | T | . | . | . | . |
| AT8 | . | . | . | T | . | . | . | . | . | . | A | . | . | - | - | - | - | - | - | - | - | - | - | - | - | . | . | . | . | . | . | . | . | . | . | . | . | . | . | . | . | . |
| AT9 | . | C | . | . | . | . | . | . | . | . | . | . | . | - | - | - | - | - | - | - | - | - | - | - | - | . | A | . | . | . | . | . | . | . | . | . | . | T | . | . | . | . |
| AT10 | . | . | . | T | . | . | G | . | . | . | A | C | . | - | - | - | - | - | - | - | - | - | - | - | - | T | . | T | . | . | . | G | A | . | . | . | . | . | . | **-** | . | G |
| AT11 | . | . | . | T | . | . | G | A | . | . | A | C | . | - | - | - | - | - | - | - | - | - | - | - | - | T | . | T | . | . | . | G | A | . | . | . | . | . | . | - | . | G |
| AT12 | . | . | . | . | . | . | . | . | G | . | . | . | . | - | - | - | - | - | - | - | - | - | - | - | - | . | A | . | . | . | . | . | . | . | . | . | . | . | . | . | . | . |
| AT13 | . | . | G | . | . | . | . | . | . | . | . | . | . | - | - | - | - | - | - | - | - | - | - | - | - | . | A | . | . | . | . | . | . | . | . | . | . | . | . | . | . | . |
| AT14 | . | . | . | T | C | . | . | . | . | . | A | . | . | - | - | - | - | - | - | - | - | - | - | - | - | T | . | T | . | . | . | . | A | A | . | . | . | . | . | - | . | G |
| AT15 | . | . | . | T | C | G | . | . | . | . | A | . | . | - | - | - | - | - | - | - | - | - | - | - | - | T | . | T | . | . | . | G | A | A | . | . | C | . | A | - | C | G |
| AT16 | . | . | . | . | . | . | . | . | . | . | . | . | . | - | - | - | - | - | - | - | - | - | - | - | - | . | A | . | . | . | . | . | . | . | A | . | . | . | . | . | . | . |
| AT17 | . | . | G | . | . | . | . | . | . | A | . | . | . | - | - | - | - | - | - | - | - | - | - | - | - | . | A | . | . | . | . | . | . | . | . | . | . | . | . | . | . | . |
| AT18 | . | . | . | . | . | . | . | . | . | . | . | . | . | **G** | **A** | **G** | **T** | **T** | **G** | **A** | **T** | **A** | **T** | **G** | **G** | . | . | . | . | . | . | . | . | . | . | . | . | . | . | T | . | . |
| **AT19** | . | . | . | . | . | . | . | . | . | . | . | . | . | **G** | **A** | **G** | **T** | **T** | **G** | **A** | **T** | **A** | **T** | **G** | **G** | . | . | . | . | . | . | . | . | . | . | . | . | . | . | . | . | . |
| **AT20** | . | . | . | T | . | . | G | . | . | . | A | C | . | **G** | **A** | **G** | **T** | **T** | **G** | **A** | **T** | **A** | **T** | **G** | **G** | T | . | T | . | . | . | G | A | . | . | . | . | . | . | G | . | G |
| **AT21** | . | . | . | T | C | . | . | . | . | . | A | . | . | **G** | **A** | **G** | **T** | **T** | **G** | **A** | **T** | **A** | **T** | **G** | **G** | T | . | T | . | . | . | . | A | A | . | . | . | . | . | G | . | G |

(-) signifies a gap

* AT not typed for in any isolate analysed in this study

Novel AT specific to the Thai population typed for in this study
