# Supplementary material for: Low Diversity Cryptococcus neoformans Variety grubii Multilocus Sequence Types from Thailand Are Consistent with an Ancestral African Origin
Source: PLoS Pathog. 2011 Apr 28;7(4):e1001343. doi: 10.1371/journal.ppat.1001343 (PMC3089418; doi:10.1371/journal.ppat.1001343)
Supplement: Table S4 — Logistic regression model best describing the prognostic factors of early death (by 10 weeks) among the Thai HIV/AIDS patients. (0.03 MB DOC) [file ppat.1001343.s004.doc]

| **Logistic regression** | **Estimate** | **Std. Error**^a^ | **z value** | **Pr(>|z|)**^b^ |
| --- | --- | --- | --- | --- |
| (Intercept) | -13.9979 | 6.7593 | -2.071 | 0.0384 * |
| Logifn^c^ | -3.4043 | 1.4931 | -2.280 | 0.0226 * |
| QCC^d^ | 1.1304 | 0.4843 | 2.334 | 0.0196 * |
|  |  |  |  |  |
| **Fisher’s Exact Test** |  | **OR**^e^ | **95% CI**^f^ | **p-value** |
| **Outcome** |  |
| GCS^g^ |  | 5.4 | 1.097 to 27.5 | 0.02 |

^a^ Standard Error; ^b^ 2-tailed p-value; ^c^ logarithmic interferon gamma; ^d^ quantitative cryptococcal cerebro-spinal fluid culture; ^e^ Odds Ratio; ^f^ confidence interval; ^g^ Glasgow Coma scale

Significance: 0 ‘***’ 0.001 ‘**’ 0.01 ‘*’ 0.05 ‘.’ 0.1 ‘ ’ 1
