# Supplementary material for: Low Diversity Cryptococcus neoformans Variety grubii Multilocus Sequence Types from Thailand Are Consistent with an Ancestral African Origin
Source: PLoS Pathog. 2011 Apr 28;7(4):e1001343. doi: 10.1371/journal.ppat.1001343 (PMC3089418; doi:10.1371/journal.ppat.1001343)
Supplement: Figure S1 — Haplotype networks of the 53 concatenated STs of the global Cng population. Sampled haplotypes are indicated by circles or rectangles colored according to the geographical region from which the sample was collected. STs unique to the African population are shown in green and consist only of clinical isolates. Haplotypes found both in Africa and elsewhere are in brown, while those not found in Africa are represented in blue. Rectangles depict the haplotype with the highest ancestral probability. Each branch indicates a single mutational difference and black dots on the lines are representative of the number of mutational steps required to generate allelic polymorphisms. Circle size is proportional to observed haplotype frequency. (0.17 MB PDF) [file ppat.1001343.s005.pdf]

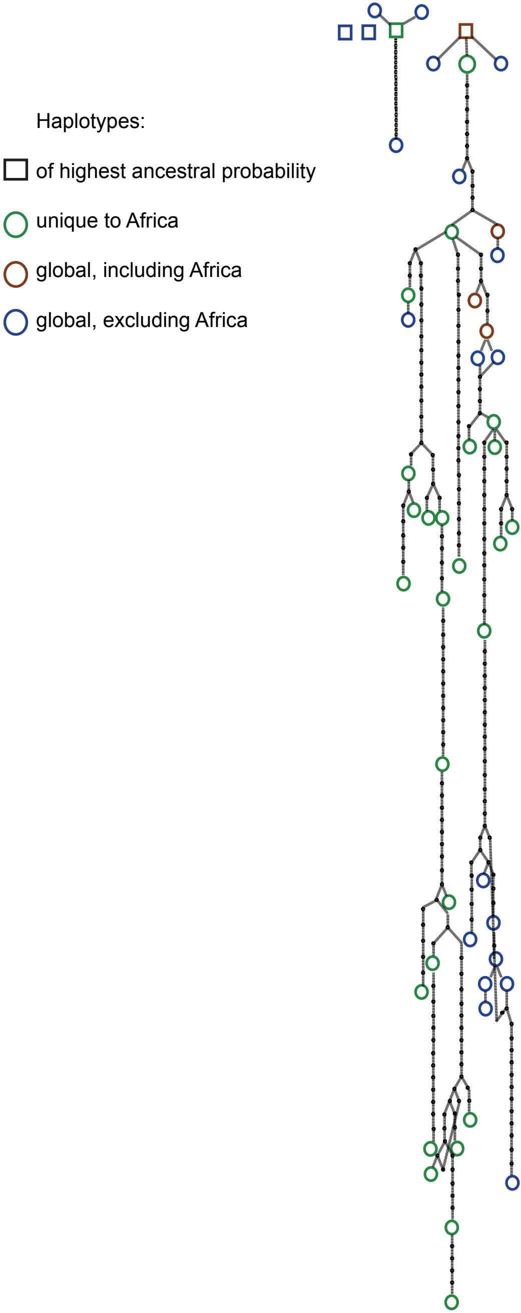
Haplotypes:
of highest ancestral probability
unique to Africa
global, including Africa
global, excluding Africa
