# Supplementary figures and images for: Low Diversity Cryptococcus neoformans Variety grubii Multilocus Sequence Types from Thailand Are Consistent with an Ancestral African Origin
Source: PLoS Pathog. 2011 Apr 28;7(4):e1001343. doi: 10.1371/journal.ppat.1001343 (PMC3089418; doi:10.1371/journal.ppat.1001343)

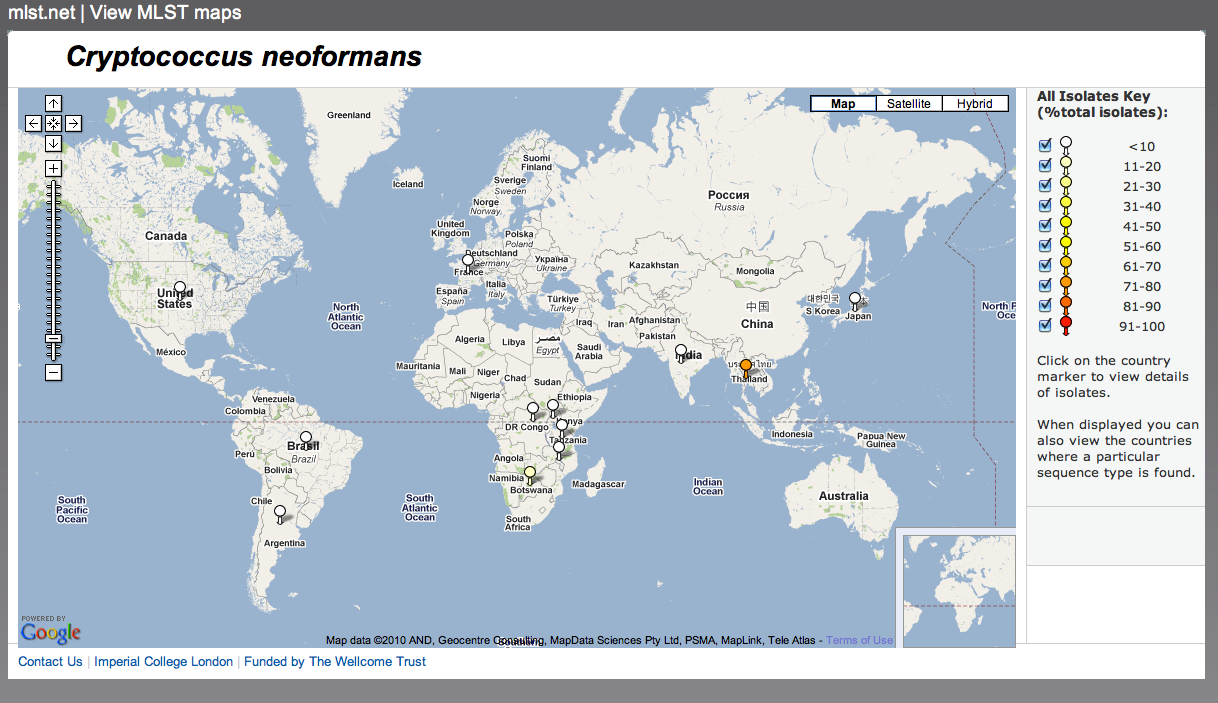

Supplement: Figure S2 — MLST map of the current global Cng isolates. This screenshot of the current distribution of Cng isolates worldwide (n = 261) depicted by the MLST website represents the mapping tool utilised in comparative eBURST analysis of Cng populations. (0.33 MB PNG) [file ppat.1001343.s006.png]
